# Prospective Medicinal Plants and Their Phytochemicals Shielding Autoimmune and Cancer Patients Against the SARS-CoV-2 Pandemic: A Special Focus on Matcha

**DOI:** 10.3389/fonc.2022.837408

**Published:** 2022-05-18

**Authors:** Caroline Joseph Kiriacos, Monika Rafik Khedr, Miray Tadros, Rana A. Youness

**Affiliations:** ^1^ Molecular Genetics Research Team (MGRT), Pharmaceutical Biology Department, Faculty of Pharmacy and Biotechnology, German University in Cairo, Cairo, Egypt; ^2^ Biology and Biochemistry Department, School of Life and Medical Sciences, University of Hertfordshire Hosted by Global Academic Foundation, Cairo, Egypt

**Keywords:** SARS-CoV-2, herbal drugs, autoimmune diseases, nutraceuticals, cancer

## Abstract

**Background:**

Being “positive” has been one of the most frustrating words anyone could hear since the end of 2019. This word had been overused globally due to the high infectious nature of SARS-CoV-2. All citizens are at risk of being infected with SARS-CoV-2, but a red warning sign has been directed towards cancer and immune-compromised patients in particular. These groups of patients are not only more prone to catch the virus but also more predisposed to its deadly consequences, something that urged the research community to seek other effective and safe solutions that could be used as a protective measurement for cancer and autoimmune patients during the pandemic.

**Aim:**

The authors aimed to turn the spotlight on specific herbal remedies that showed potential anticancer activity, immuno-modulatory roles, and promising anti-SARS-CoV-2 actions.

**Methodology:**

To attain the purpose of the review, the research was conducted at the States National Library of Medicine (PubMed). To search databases, the descriptors used were as follows: “COVID-19”/”SARS-CoV-2”, “Herbal Drugs”, “Autoimmune diseases”, “Rheumatoid Arthritis”, “Asthma”, “Multiple Sclerosis”, “Systemic Lupus Erythematosus” “Nutraceuticals”, “Matcha”, “EGCG”, “Quercetin”, “Cancer”, and key molecular pathways.

**Results:**

This manuscript reviewed most of the herbal drugs that showed a triple action concerning anticancer, immunomodulation, and anti-SARS-CoV-2 activities. Special attention was directed towards “matcha” as a novel potential protective and therapeutic agent for cancer and immunocompromised patients during the SARS-CoV-2 pandemic.

**Conclusion:**

This review sheds light on the pivotal role of “matcha” as a tri-acting herbal tea having a potent antitumorigenic effect, immunomodulatory role, and proven anti-SARS-CoV-2 activity, thus providing a powerful shield for high-risk patients such as cancer and autoimmune patients during the pandemic.

## Introduction

In October 2007, a warning letter was issued but no one responded (1). The warning letter was issued by Cheng and his colleagues mentioning that “Horseshoe bats resemble a large reservoir for SARS-CoV-like and the possibility of its reemergence with another novel virus should be taken into consideration because it is a time bomb” (1). The warning letter became a reality 12 years later in December 2019; the city of Wuhan in China experienced the emergence of a novel coronavirus that was initially called “Wuhan pneumonia” (2). It was further classified by the WHO on March 11, 2020 as the 5th documented pandemic since the 1918 Spanish flu pandemic (H1N1) (3).

SARS-CoV-2 has been the main cause of death in 2020 and 2021, accounting for more than 5 million deaths (4). Upon stratification of the mortality lists and the morbidity rates around the globe, several observations have been observed (5). Cancer and autoimmune patients such as those with asthma (6), rheumatoid arthritis (RA) (7), multiple sclerosis (MS) (8), and systemic lupus erythematosus (SLE) (7) were reported to be among high-risk patients during the pandemic (9).

In the case of cancer patients, their chemotherapy-induced immune-compromised status puts them at a higher risk to be easily infected by the virus, and at the same time, such patients should receive their treatment protocols to avoid complications from their oncological diseases (10, 11). Several reports from China (12–14), United States (15), and Italy (16–18) confirmed that cancer patients are at very high risk of developing severe complications upon SARS-CoV-2 infection. Among cancer patients, those with lung cancer are the least fortunate as it was reported that the highest incidence of comorbidity with SARS-CoV-2 was in lung cancer patients (19). Consequently, such patients experience severe symptoms of SARS-CoV-2 that may require intensive care admission and mechanical ventilation, or could result in loss of life (11). This issue encouraged oncological societies such as the European Association for Medical Oncology (ESMO) (20), the American Society of Clinical Oncology (ASCO), the National Comprehensive Cancer Network (NCCN), and many others to provide new guidelines for cancer patients’ treatment protocols and diagnostic tests during the pandemic (20). The main ideology behind the new guidelines is to calculate the benefit:risk ratio and categorize cancer patients into high, medium, and low priority based on Ontario Heath Cancer Care as previously reviewed in (21).

The same goes for patients suffering from autoimmune disorders where their immune-compromised status puts them at a higher risk of infection by the virus and developing more severe symptoms (22). In addition, their treatment protocols are mainly dependent on immunomodulatory disease-modifying therapies (DMTs) including glucocorticoids and immunosuppressants that are mainly prescribed to mitigate the immune attacks towards their normal body organs (22). For instance, a study focused on MS patients highlights that younger MS patients with lower socioeconomic status are at a higher risk of exposure to an unfavorable course of SARS-CoV-2 infection (23). In the case of SLE patients, it was first predicted that hydroxyl-chloroquine in their treatment protocol might provide a type of protection from COVID-19 complications (24). Yet, preliminary results from the clinics showed total opposite morbidity and mortality rates (25, 26). Mathian’s group reported that SLE patients also showed a high incidence of severe and even fatal cases of infection, confirming that, despite the co-treatment of SLE patients with antimalarial drugs, a high risk of unfavorable infection course has still been witnessed among SLE patients (25). Also, a more coherent study that included 417 SLE patients showed that the morbidity rates are moderately higher in the case of SLE patients (7).

Therefore, it is highly recommended that rheumatologists and oncologists encourage their patients to continue their ongoing treatment to avoid dangerous flare-ups of their autoimmune diseases or complications of their oncological diseases. It is imperative for those patients to have a nutritional plan that shields them from SARS-CoV-2 infection and at the same time improves their autoimmune status in the case of autoimmune patients and/or provide antitumor actions in the case of cancer patients.

In this review, we will show a glimpse of all the therapeutical trials that were carried out during the last couple of years to decrease the socioeconomic burden of such a pandemic. Yet, several failed attempts were witnessed starting from repurposing of conventional drugs, discovery of new medications that might take years of validation, to several vaccination approaches that go in parallel with the high viral mutational capacity (27, 28). However, less attention was given to the ideal remedy—”herbal drugs”—that might be the ultimate route to treat such deadly disease.

In this review, the authors will try to emphasize the significance of herbal drugs that should not be less than that of vaccines and antivirals during the pandemic. Herbal drugs have an edge regarding high-risk patients (cancer and autoimmune patients) in that they might play a dual/triple role in alleviating the primary disease and act as a protective shield during the pandemic.

Upon focusing on the herbal products with their immense roles starting from being antioxidants and holding anti-inflammatory and antiviral activities, we had a closer look at “matcha”, which we expect to have a great impact in the upcoming years because of its potent immune-modulatory capabilities and its recent validated activity against SARS-CoV-2 (29, 30). Nonetheless, matcha was also reported to hold a lot of promise for cancer (31, 32) and autoimmune patients (33, 34). Yet, in this review, the authors shed light on the research gap concerning the molecular mechanism of actions underlying matcha as a potent immunomodulatory, anticancer, and antiviral activity.

## Methodology

In this review, the authors screened literature covering the therapeutic effects of “matcha” as a SARS-CoV-2 antiviral herbal drug; this review also focused on the anticancer activity and immunomodulatory role of “matcha”. To attain the purpose of the review, research was conducted at the States National Library of Medicine (PubMed). For the search in databases, the descriptors used were “COVID-19”/“SARS-CoV-2”, “Herbal Drugs”, “Autoimmune diseases”, “Rheumatoid Arthritis”, “Asthma”, “Multiple Sclerosis”, “Systemic Lupus Erythematosus” “Nutraceuticals”, “Matcha”, “Green tea”, “EGCG”, “Quercetin”, “Cancer”, and key molecular pathways. Research papers, books, and published data were reviewed for their relevance to the aim of the review and summarized. Criteria for inclusion were complete, relevant publication, available online, in English, published between 1997 and 2022, and with detailed information about participants, methods, and analyses. Data collection was performed, and data abstracted were in the form of descriptive information, covering the type of samples used, techniques, and findings or effects reported. Bias was limited through the evaluation of the studies through their internal validity rather than the conclusion.

### SARS-CoV-2 Structure and Life Cycle

**Figure 1:**
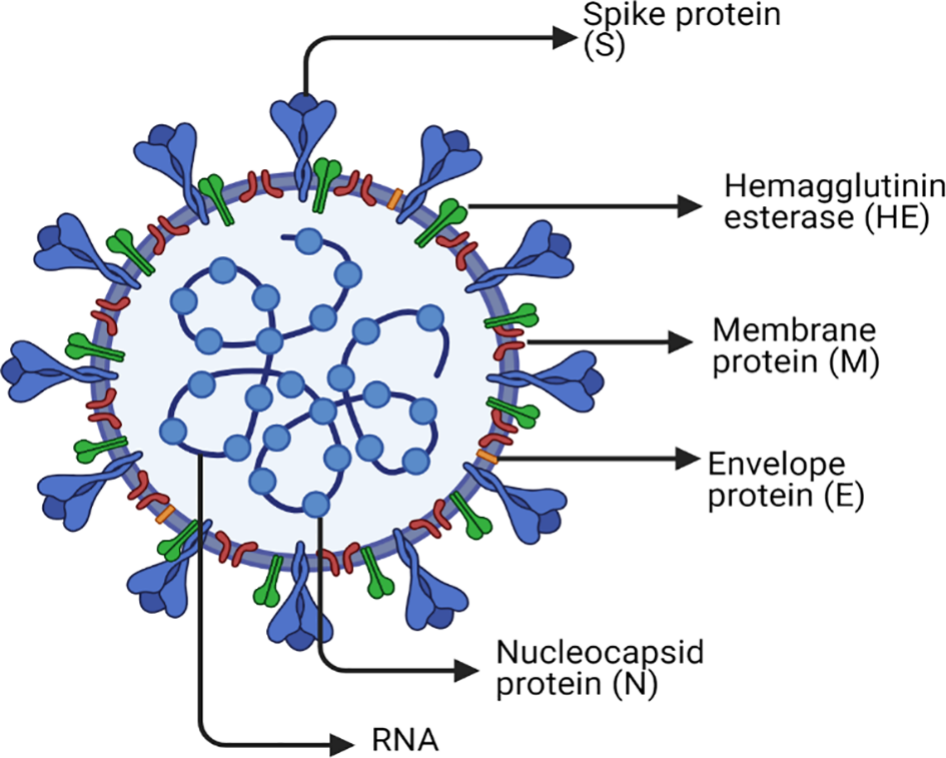
SARS-CoV-2 structure. The figure represents a graphical representation of the viral structural proteins spike (S), envelope (E), and membrane (M), which are embedded in the lipid surface. The positive single-stranded RNA is bound to the nucleocapsid protein (N) in the core of the capsid. Each one of these proteins plays a crucial role in the replication life cycle of the virus. The spike protein (S) is the master that supports the attachment and entry of host cell *via* fusion. The nucleocapsid protein (N) is the one used in transcription, which is included in the replication cycle. The membrane protein (M) that is most abundant on the viral surface drives the viral assembly. Furthermore, the envelope protein (E) has an indispensable role in assembly, host cell membrane permeability, and interactions between the host and virus. Another surface protein is Hemagglutinin esterase dimer (HE) that is found to play a role in cell entry and its infection without having a role in the replication process itself. Finally, the lipid envelope encircles the approximately 30,000 nucleotides, which is the genome of the virus encoding its four structural and many nonstructural proteins (nsp).

**Figure 2:**
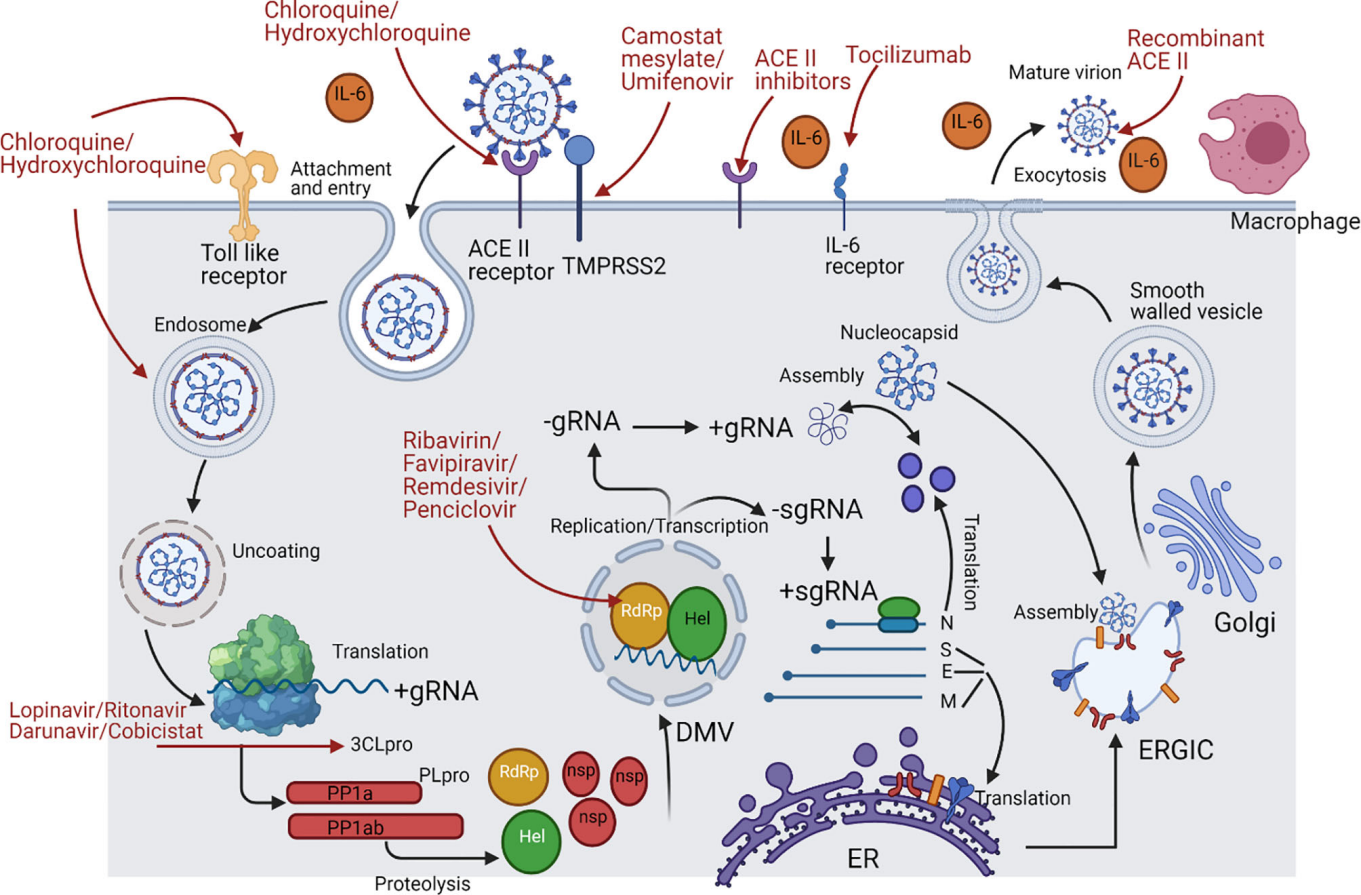
SARS-CoV-2 life cycle and the repurposed drugs targeting specific stages throughout its life cycle. This figure represents a schematic description for the SARS-Cov-2 life cycle with the repurposed drugs targeting specific stages in it. ACE2 receptor on the lung cells is targeted by the RBD of the S1 region in the viral spike protein; however, this binding could be targeted by chloroquine and hydroxychloroquine, recombinant ACEII receptor, or ACEII inhibitors. After the attachment, transmembrane protease serine 2 (TMPRSS2) of the host cell makes a proteolytic cleavage between S1 and S2 subunits, thus separating RBD from the fusion domains, yet this step could be targeted by camostat mesylate and Umifenovir. Consequently, a major step is taken, which is the exposure of fusion peptide domain, enabling the virus to fuse with the cell and pave its way by endocytosis then enclosed in an acidified endosome. Proteasomes then act on the nucleocapsid protein (N), uncoating it and releasing the genetic material freely in the cytoplasm, but this can be inhibited by chloroquine and hydroxychloroquine due to rendering alkaline endosomal PH. Once the positive strand becomes free, translation of the open reading frame 1a/b and production of polyproteins pp1a and pp1ab takes place. The polyproteins undergo cleavage by the viral proteases Papain like protease (PLpro) and Chemotrypsin like protease (3C like protease or 3CLpro or Mpro). Lopinavir/Ritonavir and Darunavir/Cobicistat are the ones used to inhibit 3CLpro. On the other hand, the transcription process start since the Replication/Transcription complex (RTC) was translated, and at this point, there are many nucleoside analogue drugs that were repurposed for inhibiting RNA-dependent RNA polymerase (RdRp) such as Ribavirin, Favipiravir, Remdesivir, and Penciclovir. The RTC will supervise the formation of double membrane vesicle structures (DMV) in the cytoplasm to shield the transcription process. The positive strand is used as a template for making the negative strand, which is then transcribed to make more positive strands. Moreover, subgenomic mRNAs are produced by discontinuous transcription for the sake of being translated to form the 4 viral structural proteins. Once N protein is finished, it combines with a new positive strand for the nucleocapsid to be done. However, S, E, and M proteins proceed to the endoplasmic reticulum (ER) and then to the Golgi apparatus. Last but not least, both the nucleocapsid and structural proteins will be assembled at the ER-Golgi intermediate compartment (ERGIC) to the viral envelope followed by exocytosis of mature virions through smooth-walled vesicles. Many immune components can be released during the whole process such as IL-6, leading to a cytokine storm, so the monoclonal antibody Tocilizumab is used as well as the Toll-like receptor (TLR) inhibitors chloroquine and hydroxychloroquine.

SARS-CoV-2 has a spherical shape with a positive single-strand RNA composed of approximately 30,000 nucleotides and enclosed inside a capsid (35). The genome encodes four structural proteins and many non-structural proteins (nsp) as previously reviewed (36, 37). The structural proteins are Spike (S) protein, Envelope (E) protein, Membrane (M) protein, and Nucleocapsid (N) protein. Inside the capsid, there is a nuclear capsid or the N protein, which is bound to the positive single-stranded RNA and coating it as demonstrated in **Figure 1**. The SARS-CoV-2 life cycle is briefly described in **Figure 2**, since it has been extensively discussed and reviewed in previous reviews (35, 38).

### Failed Trials to Handle the Pandemic

The current pandemic has urged the public health systems and pharmaceutical companies to develop new antiviral drugs and vaccines against SARS-CoV-2 after being the leading cause of death recently. In an attempt to find effective treatment for COVID-19 patients, enormous efforts were exerted in handling the pandemic. Several approaches were considered such as repurposing of FDA-approved drugs where the doctors were permitted to carry out such clinical trials using a combination of these drugs due to the urgent need to reduce cost, time, and risk of the drug development processes, but this was accompanied by several side effects and limitations as shown in **Table 1**. Thus, not all the repurposed drugs have been approved to be used in ameliorating this pandemic, and some of them were suspended by WHO such as chloroquine, hydroxychloroquine, remdesivir, and lopinavir/ritonavir (74). It is also important to note that all clinical trials highlighted in **Table 1** do not include any of the high-risk patients like cancer and autoimmune patients, who are the main concern of this review.

**Table 1:** A list of currently available drugs for the treatment of SARS-CoV-2.

Drug	No. of Clinical Trial	Clinical Trial Status	No. of Participants	Participants’ Average Age	Participants’ Status	Intervention	Results	Side Effects	Limitations	Ref.
**Ribavirin**	NCT04551768	Completed	51	>18 years	Hospitalized	-50 mg/ml over 1 h, 2 times/day for 6 days -100 mg/ml over 30 min 2 times/day for 6 days	Pending	-Hemolytic anemia -Fatigue -Skin Rash -Leukopenia -Teratogenicity	-Excision ability of false nucleotides acquired to coronaviruses by nsp14	(39–42)
**Favipiravir**	NCT04694612	Completed (Published Results)	30	52.5	Hospitalized	Baloxavir/Marboxil + existing treatment: 80 mg/day on days 1 and 4 Favipavir + existing treatment: 1,600 or 2,200 mg then 600 mg 3 times/day Control: existing treatment	-No extra advantage on using Favipiravir -More vigorous clinical trials are needed to be approved for its international use	-Elevation of liver enzymes -Nausea -Vomiting	-Safety concerns about QT prolongation -Teratogenic potential is unclear	(39, 43–45)
**Remdesivir**	NCT04280705	Completed (Published Results)	1,062	≥18 years	Hospitalized	Remdesivir or Placebo, 200 mg IV on day 1 then 100 mg once/day for 10 days	No notable benefit specially for mild to moderate diseased patients at day 28	-Worsened respiratory problems -Nausea -Constipation -High alanine aminotransferase levels	-High mortality rate -Not sufficient as sole antiviral treatment -No improvement in mortality, time of virus clearance or time of clinical improvement	(46–48)
	NCT04871633	Completed	66	>12 years	Hospitalized	Remdesivir IV 200 mg followed by 100 mg/day for 5–10 days	Pending	N/A	N/A	
**Camostat mesylate**	NCT04321096	Recruiting	580	≥18 years	Hospitalized/Outpatients	Placebo: 2 pills 3 times/day for 5 days Camostat mesylate: 2 × 100 mg pills 3 times daily for 5 days	Pending	-Skin rash -Pruritus -Abdominal discomfort -Elevation of liver enzymes	-Early treatment initiation at the first phase of infection is needed	(49–51)
	NCT04608266	Recruiting	596	≥18 years	No initial hospitalization requirement	Placebo: 2 tablets every 8 h for 14 days Camostat mesylate: 2 × 100 mg tablets every 8 h for 14 days	Pending	N/A	N/A	
**Darunavir**	NCT04252274	Recruiting	30	Child, Adult, Older adult	N/A	Darunavir and cobicistat one tablet/day for 5 days + conventional treatments Conventional treatment only	Pending	-Increasing risk of cardiovascular diseases with increased exposure	-Well established pharmacological profile is needed	(49, 52, 53)
**Lopinavir/Ritonavir**	NCT04252885	Completed	86	18–80 years	N/A	-Lopinavir (200 mg) and ritonavir (50 mg) 2 tablets each, q12h, for 7-14 days +standard therapy -Arbidol (2 × 100 mg) tid, for 7-14 days +standard therapy -Standard treatment	Pending	-Diarrhea -Nausea -Asthenia	-Further safety profile is needed -No significant improvement in mortality rate, viral load reduction or on clinical level	(54)
**Chloroquine/Hydroxychloroquine**	NCT04322123	Active; not recruiting	630	≥18 years	Hospitalized	Hydroxychloroquine 400 mg BID for 7 days -Hydroxychloroquine 400 mg BID + azithromycin 500 mg once a day -Standard treatment protocol	Pending	-Cardiac arrest -QT prolongation -High rate of hospital deaths	-Narrow therapeutic index -Cardiac toxicity -Safety and efficacy proofs are needed	(55–59)
**Nitazoxanide**	NCT04486313	Completed	1,092	12–120 years	N/A	Nitazoxanide, 2 × 300 mg tablets BID with food for 5 days -Placebo, 2 tablets BID with food for 5 days	Pending	-Abdominal cramps -Diarrhea	-Further evidence is required or hepatorenal -Cardiac toxicity -Teratogenic effects	(60)
**Umifenovir**	NCT04260594	Completed	236	18–65 years	N/A	-Arbidol 2 tablets, tid for 14–20 days -Ordinary treatment	Pending	-Nausea -Vomiting	-Limited safety and efficacy documents	(61)
**ACE II inhibitors**	NCT04364893	Recruiting	700	≥18 years	Hospitalized	- ACEI/ARBs discontinuation - ACEI/ARBs continuation	Pending	-Inflammatory lung diseases -Impaired lipid and glucose metabolism -Cardiac toxicity -Renal malfunction -Impaired immunity	-Negative impact on associated comorbidities as diabetes and hypertension	(62–64)
**Recombinant ACE II**	NCT04382950	Not yet recruiting	24	18–60 years	N/A	-rbACE2 IV 0.4 mg/kg BID for 7 days + Aerosolized 13 cis retinoic acid from 0.2 mg/kg/day to 4 mg/kg/day -Standard therapy	Pending	-Hypotension -Acute kidney injury	-More preclinical and clinical studies are still needed -Effective only at the early stage of infection	(65, 66)
**Convalescent Plasma**	NCT 04343261	Completed/Has results	48	18–90 years	N/A	2 convalescent plasma infusions (2 × 200 ml) each for 1 h	-No significant change in number of days required to discharge, between testing positive and receiving plasma and same for those who died	N/A	-No improvement for critical cases -Finding suitable donors -Disease transmission risk	(67–70)
**Tocilizumab**	NCT04356937	Completed/Has results	243	18–85 years	Hospitalized	-IV at dose 8 mg/kg + standard therapy - IV at dose 4 mg/kg + standard therapy	-Failure in death or intubation prevention in moderately ill hospitalized patients	-Septic shock -GIT perforations -Leukopenia -Lymphopenia	-High cost -Opportunistic infections risk -More studies are required	(39, 71–73)

Throwing light on the currently available vaccines’ effectiveness, it was reported that only 30.7% protection was acquired against the new variants of concern “delta” when compared to the “alpha” variant of the virus, which has provided 48.7% protection from a single dose of either BNT162b2 or ChAdOx1 nCoV-19 vaccines (75). However, two doses from these vaccines give a 93.7% protection against alpha and 88% protection against delta for BNT162b2, while for ChAdOx1 nCoV-19, it has an efficacy of 74.5% against alpha vs. 67% for delta (76). For the Pfizer/BioNTech vaccine efficiency, it has 88% protection against the alpha variant, and this percentage has significantly decreased against delta (76). Nonetheless, it was reported that certain mutations were identified in the most recent “omicron” variant that led to higher transmission ability, higher infectivity and binding affinity to ACE2 receptors, and increase in the failure of neutralizing antibodies and immune defense (77). Therefore, relying only on the significance of vaccinations to rescue us from such virulent variants is not a wise solution, especially since it has been well documented and experienced that the vaccines developed against the wild SARS-CoV-2 have lower efficiency rates against the mutated variants (78). Collectively, it has to be recognized that at this stage, vaccine development is important, but still the nourishment of our immune systems has a greater weight in fighting this ongoing pandemic.

After shedding light onto the evolution of new variants of SARS-CoV-2, it is essential to recall the long-lasting Influenza A virus as a live example, which can be compared in parallel with SARS-CoV-2 nowadays. The Influenza A virus causes one of the annual epidemics; even so, it continues to represent a significant threat to global public health due to its very high mutation rates and its ability to cross-transmit between species (79). The same scenario applies where Influenza A rapid evolution resulted in the loss of optimal efficacy for vaccines and antiviral drugs, to which the virus became resistant and thus complete eradication was not achieved (79). As a result, the scientific communities were compelled to use natural therapies and herbal products to boost the immune system as an alternative plan, which showed great success. Some of these herbal products include licorice roots, pomegranate, guava tea, vitamin C supplements, and zinc supplements (80).

### Current Status of Cancer Patients and Autoimmune Patients After the Emergence of SARS-CoV-2 Variants of Concerns

Focusing on cancer patients and patients with autoimmune diseases, there are several studies that showed impaired antibody responses following dual COVID-19 vaccination in patients with chronic lymphocytic leukemia (81) and lung cancer (82). Furthermore, it was proved that humoral protection against the delta variant is markedly impaired among chronic lymphocytic leukemia patients, indicating the urgent need for further optimization of immune protection in this patient cohort (81). Yet, not enough data were reported about the status humoral protection for patients with autoimmune diseases.

### Herbal Drugs: From Passenger to Driver Seat During the Pandemic

Applying the concepts of ancient people about natural remedies in defending against colds and flu ensures that natural products were always side by side with any respiratory viral infection (83, 84). By now, most of the population had experienced the impaired protection of the currently available drugs against SARS-CoV-2 and vaccines due to the high rate of naturally occurring mutations. Consequently, a noteworthy concept is that we need an immune-modulatory and broad-spectrum antiviral agent with diverse mechanisms of action that can be readily used for the prevention of future pandemics. In this review, the authors will focus on candidates from herbal medicines exerting their immunomodulatory and antiviral effects especially for immune-compromised COVID-19 patients, and a special focus on the Japanese green tea “matcha” will be addressed. It is also worth mentioning that several reviews had shed light onto the potent role of natural compounds in the prevention of and/or as an adjunct treatment for COVID-19 (85–88). Yet, this review focuses on the tri-acting natural compounds that possess anticancer, immunomodulatory, and anti-SARS-CoV-2 activities, which were proposed as protective shields for cancer and autoimmune patients in particular during the pandemic.

### Candidates from Herbal Medicine During the Pandemic

In this section, the authors will focus on candidates from the herbal medicine field that have been suggested to be used during the pandemic. During the last couple of years, a huge number of herbal medicines have been suggested as anti-SARS-CoV-2 agents, for example, purple coneflower, the bark of cinchona trees, Java turmeric, ashwagandha leaves, ginger, turmeric, garlic, flaxseed, tick berry leaves, oregano, elderberry, green tea, orange, and citrus peel as previously reviewed in (89, 90).

This review focuses on natural compounds that possess a triple action including anticancer, immunomodulatory, and anti-SARS-CoV-2 activities as listed below and as summarized in **Table 2**. The inclusion criteria include natural compounds that possess the 3 activities, with a known mechanism of action and molecular targets, and entered clinical trials in the case of anti-SARS-CoV-2 herbal drugs. A detailed list of natural compounds that entered clinical trials as anti-SARS-CoV-2 agents is shown in **Table 3**. The exclusion criteria used in this review include natural compounds that possess only one of the above-mentioned actions, and/or unknown mechanism of action.

**Table 2:** Tri-acting (anticancer, immunomodulatory, and anti-SARS-CoV-2) natural products.

Natural Product	Active Constituent	Anti cancer Mechanism of Action	Immunomodulatory Mechanism of action	Anti-SARS-CoV-2 Mechanism of action	Ref.
**Ginger**	6-gingerol 6-shogaol 10-gingerol	-Induction of apoptosis by increasing caspase-3/7 in gastric cancer cells -Downregulation of cytosolic inhibitor of apoptosis (cIAP)-1 in gastric cancer cells -Inhibition of TRAIL-induced nuclear factor-kappaB (NF-κB) activation in gastric cancer cells	-Inhibiting the expression of inducible nitric oxide synthase (iNOS) and cyclooxygenase-2 (COX-2) in macrophages in multiple sclerosis -Inhibiting LPS-induced NO and production of pro-inflammatory cytokines by inhibiting the NF-kB activation in BV2 microglial cells in multiple sclerosis	-Inhibition of binding between S protein and ACE2 *in silico*	(91–95)
**Turmeric**	Curcumin	-Upregulation of miRNA-192-5p and suppression of PI3K/Akt signaling pathway in non-small cell lung cancer	-Reduced levels of pro-inflammatory cytokines (TNF-α and IL-1β) in the serum and synovial fluid in adjuvant-induced arthritis in rats -Regulates the cyclooxygenase (COX) and lipoxygenase (LOX) enzymes, leading to the suppression of various pro-inflammatory mediators, including MMP9 and MMP13 in arthritis -Inhibition of IL-12 signaling pathway in T cells in multiple sclerosis.	-Inhibition of Toll-like receptors, NF-κB, inflammatory cytokines and chemokines, and bradykinin, decreasing SARS-CoV-2 symptoms	(96–104)
**Garlic**	Diallyl disulfide (DADS) Alliin	-Proapoptotic effect by histone deacetylation, inhibition of ERK, activation of SAPK/JNK, and p38 pathways in MCF-7 breast cancer cells.	-Suppression of LPS inflammatory signals by generating an anti-inflammatory gene expression and preventing the increase in expression of pro-inflammatory cytokines IL-6 and MCP-1 in LPS induced inflammation in 3T3-L1 adipocytes.	-Attenuation of coronavirus infection by dual S-thioallylation of SARS-CoV-2 Mpro *in silico*	(105–113)
**Flaxseed**	Omega 3	Upregulation of BAX, downregulation of Bcl-2 and increase in DNA fragmentation in acute myeloid leukemia.	-Reducing the level of antibodies (anti-dsDNA), interleukins (IL-1α, IL-1β, and IL-2) and TNF-α in systemic lupus erythematous.	-Incorporation of Omega-3 in phospholipid bilayer of cell membranes leading to production of less pro-inflammatory mediators.	(114–116)
**Citrus fruits**	Hesperidin	-Induction of tumor suppressor miR-486-5p and repression of oncogenic long non-coding RNA H19 in breast cancer -Repression of metastatic mediator ICAM-1 in breast cancer	-Suppression of the levels of IL-4, IL-5, IL-13, and IgE levels in serum in mouse model for asthma -Increase the Treg cells production of interleukin IL-10, transforming growth factor (TGF-β), reduction in production of IL-17 and IL-6, decrease in the percentages of Th17 cells, % of Treg cells in the spleen and lymph nodes, reduces ROR-γt factor expression, but enhanced Foxp3 expression in mouse model for multiple sclerosis	-Binding to the TMPRSS2 and ACE2 and block the viral entry *in silico*	(117–120)
**Black tea**	Theaflavins Theaflavin-3, 3’-digallate (TFDG)	-Reduction in tumor-induced angiogenesis by downregulation of VEGF and HIF-1a in ovarian cancer cells	-Inhibiting the activation of NF-κB- and MAPK-signaling pathways in Rheumatoid arthritis.	-Blockage of viral RNA-dependent RNA-polymerase by *in silico* docking.	(121–125)
**Green tea**	EGCG Quercetin	-Decrease Bcl-2 expression, increase expression of caspase 3 and Bax in esophagus cancer -Induction of apoptosis and downregulation of PI3K, PKC, COX-2, and ROS. Increased expression of p53 and Bax in liver cancer.	-Reducing IgE and histamine levels, Decreasing FcϵRI expression, regulating the balance of Th1/Th2/Th17/Treg cells and inhibiting related transcription factors in asthma.	-Inhibition of Mpro by *in silico* studies -Inhibition of 3CLpro and PLpro by *in silico* studies	(126–132)
***Tripterygium wilfordii* Hook. F.**	Extract Triptolide	-Accumulation has a small of p53 and apoptotic cell death in human prostatic epithelial cells - The sensitivity of gemcitabine-resistant cells to cisplatin treatment is enhanced by activation of mitochondria-initiated cell death pathway and suppression of HSP27 expression in pancreatic cells	NF-κB, NF-κB/TNF-α/vascular cell adhesion molecule-1, and TGF-β1/α-smooth muscle/vimentin signaling pathways induced by TNFs and TLR4 in rheumatoid arthritis -Downregulation of p38 MAPK and NF-κB signaling pathways in neuroinflammation	N/A	(133–136)
***Eucalyptus globulus* Labill.**	Extract 1,8-Cineol	-Suppresses the proliferation of human colon cancer cells by inducing apoptosis	-Reduces the expression of NF-κB target gene MUC2 in asthma	-Inhibition of Mpro *in silico.*	(137–139)

**Table 3:** Herbal drugs in clinical trials against SARS-CoV-2.

Naturalcompound	Type	Dosage form	Clinical Trial No.	Results	Dose	Mechanism of action	Therapy type	Phase	No. of participants	Clinical trial state	Ref.
Echinacea purpurea	Nutraceuticals	Tablets (Echinaforce)	NCT05002179	Pending	Prevention: 800 mg 3 times/day Treatment: 800 mg 5 times/day	N/A	Primary therapy	Phase IV	122	Completed	(140)
Ashwagandha, Giloy, and Tulsi combination (Ayurveda Intervention)	Traditional medicine	Tablets	NCT04716647	Pending	Ashwagandha: Doses range from 250 mg to 5 g Giloy: Doses range from 500 mg to 1 g Tulsi: Doses range from 500 mg to 1 g	- Inhibitor of the main protease (Mpro or 3Clpro) - Inhibition of the TMPRSS2/ACE II complex	Primary therapy	N/A	28	Completed	(141, 142)
Turmeric	Nutraceuticals	Tablets (NASAFYTOL)	NCT04844658	Pending	1008 mg 8 times/day	- PLpro inhibitor	Supportive therapy	N/A	51	Completed	(89, 143)
Psidii Guava’s	Herbal extract	Capsules	NCT04810728	Pending	2 caps 3 times/day	- Inhibitor of 3CLpro and PLpro (mainly quercetin)	Primary therapy	Phase III	90	Completed	(141)
Flaxseed	Nutraceuticals (omega3 fatty acid)	N/A	NCT04836052	Pending	2 mg 2 times/day	- Attenuate pro-inflammatory cytokines	Primary therapy	Phase III	372	Recruited	(144)
Hesperidin	Bioactive phyto-compound	Capsules	NCT04715932	Pending	500 mg 2 times/day	- Inhibitor of 3CLpro and PLpro	Primary treatment	Phase II	216	Completed	(145)
Ginger	Nutraceuticals	Tablets	IRCT20200506047323N1	Pending	1,000 mg 3 times/day	- Inhibitor of PLpro	Primary treatment	Phase III	86	Completed	(146, 147)
Green tea	Nutraceuticals	Capsules	IRCT20150711023153N3	Pending	450 mg 2 times/day	- Inhibitor of 3CLpro and PLpro -Inhibits complex formation with the virus	Supportive therapy	N/A	74	Completed	(30, 148)
EGCG	Nutraceuticals	Capsules	NCT04446065	Pending	250 mg	- Inhibitor of 3CLpro and PLpro -Inhibits complex formation with the virus	Primary treatment (prophylaxis)	Phase II	524	Not yet recruiting	(30, 148)
Colchicine	Bioactive metabolite	Tablets	NCT04363437	Pending	An initial dose of 1.2 mg followed by 0.6 mg after 2 h on day 1. After that, 0.6 mg of two doses up to the 14th day	- Disruption of microtubules and thus affect viral trafficking and the formation of double-membrane viral vesicles	Primary therapy	Phase II	70	Recruiting	(149)
Quercetin	Bioactive metabolite	Tablets	NCT04377789	Pending	500 mg of quercetin given daily to the prophylaxis group. The quercetin treatment group had confirmed cases of COVID-19 and they were provided with 1,000 mg quercetin daily.	- Inhibition of polymerases, proteases, and reverse transcriptase; suppressing DNA gyrase; and binding viral capsid proteins; thus, it possesses an effective antiviral activity	Primary therapy	N/A	447	Completed	(132)
Escin	Nutraceuticals	Tablets	NCT04322344	Pending	Oral administration of standard therapy Escin tablet for 12 days (40 mg thrice a day)	- Potent antiviral activity. Yet, the exact mechanism of action is still unknown.	Adjuvant therapy	Phase II/III	120	Recruiting	(150)
Nicotine	Bioactive phytocompound	Patches	NCT04608201	Pending	As Nicotine patch 0.5 patch for day 1 and day 2 1 patch for day 3 and day 4 1.5 patches for day 5 and day 6 2 patches from day 7 to the day of discharge from hospital (Each patch contains 7 mg nicotine)	- Inhibits the penetration and spread of the virus - Prophylactic effect in COVID-19 infection	Primary therapy	Phase III	220	Recruiting	(151)

#### Shufeng Jiedu

Shufeng Jiedu capsule (SFJDC) is an oral Chinese herbal medicine prepared from many different plants as rhizome and root of *Polygonum cuspidatum*, root of *Isatis indigotica* Fort, dried roots of *Phragmites communis*, and many others (53, 152). SFJDC was proven to have antibacterial, antiviral, anti-inflammatory, and antitumor effects (153). The capsule preparations are often used to cure Influenza, the thing that made these preparations to be suggested for investigating it against COVID-19 (53). Yet, it is worth mentioning that SFJDC is contraindicated in patients with known serious hypersensitivity to the product itself or any component of the dosage form.

##### Anticancer Activity of SFJDC

In a study held to discover the effects of combining SFJDC with doxorubicin to treat hepatocellular carcinoma cells, results showed higher incidence of apoptosis along with more inhibition in cancer migration and invasion, indicating that SFJDC could be a potential complementary anticancer medication (153).

##### Immunomodulatory Role of SFJDC

The anti-inflammatory action of SFJDC was studied using mouse models infected with HCoV-229E, and the study indicated the ability of SFJDC to decrease IL-6, IL-10, TNF-α, and IFN-γ in lungs. This has created the hypothesis about the ability of herbal medicines to attenuate the cytokine storm caused by COVID-19 (154). These effects could be explained by the following mechanisms in which SFJDC was found to be acting with them: the PI3K-Akt signaling pathway was attenuated and the NF-κB-mediated transcription of pro-inflammatory cytokines was inhibited as well (155, 156).

##### Anti-SARS-CoV-2 Activity of SFJDC

The main constituents of SFJDC are quercetin, wogonin, and polydatin, indicating their ability to bind to Mpro of SARS-CoV-2 by means of molecular docking studies (157). There are clinical data as well for the addition of SFJDC with the standard antiviral therapy, indicating the high probability of SFJDC to shorten the duration of COVID-19 symptoms in mild to moderate cases (157). Another study was conducted at Bozhou People’s hospital where the effect of combining SFJDC with arbidol hydrochloride was studied in comparison to the arbidol hydrochloride alone (158). The results revealed clinical improvements in the combined group compared to the other one (152, 159).

#### Ginger

*Zingiber officinale* or ginger belongs to the family Zingiberaceae, it is an extremely beneficial herbal medicine used in many aspects. It originated in Southeast Asia but nowadays used worldwide as a food spice (160). Ginger rhizome is used for pain, nausea, and vomiting (161). A very wide range of active constituents are available and divided into two groups: volatile and non-volatile. The volatile group is definitely responsible for the odor and taste of ginger such as sesquiterpene and monoterpenoid hydrocarbons. However, gingerols, shogaols, parasols, and zingerone are the non-volatile constituents (162). Yet, it is worth mentioning that the usage of ginger might be accompanied by several side effects such as abdominal discomfort, diarrhea, heartburn, increased bleeding tendency, and mouth or throat irritation.

##### Anticancer Activity of Ginger

Ginger’s active constituents 6-gingerol and 6-shogaol are the main anticancer agents. Ginger has a broad spectrum anticancer activity against an array of solid malignancies such as gastric, pancreatic, colorectal, and liver cancers as shown in **Table 2** and as previously reviewed in (91). The anticancer activity of ginger is accredited to its aptitude to repress several signaling pathways simultaneously such as the PI3K/AKT/mTOR pathway, the JAK/STAT pathway, the NF-κB pathway, COX-2 signaling, and caspase molecules (91).

##### Immunomodulatory Role of Ginger

Ginger is now considered a perfect choice for COVID-19 patients as it has analgesic, anti-inflammatory, antiviral, and immunomodulatory effects that can have a great role in the prevention of lung damage and respiratory disorders as listed in **Table 2**. Mechanistically, this analgesic effect is achieved by inhibiting prostaglandin (PG) production through cyclooxygenase (COX) and lipoxygenase (LOX) pathways. This is also achieved by its antioxidant activity where inhibition of the transcription factor Nf-ĸB occurs. It also acts as an agonist of vanilloid nociceptor, which represses the pain sensation (163). Considering the anti-inflammatory effect, several pathways are involved, but we will focus only on the effect of 6-gingerol, which inhibits the production of pro-inflammatory cytokines from LPS-stimulated macrophages as shown in **Table 2** (164). In the case of immune-compromised patients such as patients with rheumatoid arthritis, its manifestations are proved to be decreased by ginger as it increases the transcription factor forkhead box protein 3 (FoxP3) gene expression and decreases retinoic acid receptor-related orphan receptor γt (RORγt) and T-box expressed in T-cell (T-bet) gene expression (165).

##### Anti-SARS-CoV-2 Activity of Ginger

As illustrated earlier, one of the drug targeting mechanisms for SARS-CoV-2 is a papain-like protease (PL pro) that cleaves viral polyproteins that are very important for viral replication and survival. It was recently reported that ginger has the potential to act as a PL pro inhibitor for SARS-CoV-2, expressing its antiviral effect (89). Nonetheless, ginger has proven to relieve symptoms associated with COVID-19 infection such as chest pain. Ginger has proven to reduce chest pain and induce relaxation in airway smooth muscle, hindering airway resistance and inflammation as shown in **Table 3** (166).

#### Turmeric

*Curcuma longa* or turmeric is a widely known herbal medicine; its main active constituent is the polyphenolic compound curcumin. It belongs to the family Zingiberaceae and used as a food spice, same as ginger. In Asian countries, it is used as a supplement and medicine to treat many diseases such as diabetes mellitus, cardiovascular diseases, obesity, neurodegenerative diseases, inflammatory bowel disease, allergy or asthma, and psoriasis. As mentioned above, turmeric extract is known for its polyphenol curcumin, constituting up to 77%; it contains other active constituents such as demethoxy-curcumin and bis-demethoxy-curcumin (167, 168). Turmeric can be used as an antiviral, antioxidant, anti-inflammatory, and anticancer agent. It is also important to note that turmeric does not usually cause severe side effects. Some users experience mild side effects such as abdominal discomfort, nausea, diarrhea, and dizziness.

##### Anticancer Activity of Turmeric

Turmeric is one of the well-investigated anticancer nutraceuticals. It was named the golden spice, whose use was passed on from the kitchen to the clinic (169). Curcumin shows an anti-neoplastic activity against solid malignancies such as breast, liver, colorectal, and prostate cancers, and several types of leukemias and lymphomas as shown in **Table 2** and previously reviewed in (170–172).

##### Immunomodulatory Role of Turmeric

Turmeric is ranked as one of the most common immunomodulatory herbal drugs as curcumin shows strong antioxidant and anti-inflammatory effects (173). Mechanistically, its anti-inflammatory effects are prominent through the inhibition of the pro-inflammatory molecules: toll-like receptor (TLR-4), phosphatidylinositol-3 kinase (PI3K), and nuclear factor-kappa B (NF-*κ*B). Turmeric also has the potential to repress the production of an array of pro-inflammatory cytokines such as IL-6, tumor necrosis factor-alpha (TNF-*α*), and interleukin 1 beta (IL-1*β*) (174, 175).

##### Anti-SARS-CoV-2 Activity of Turmeric

Concerning the SARS-CoV-2 antiviral activity of turmeric, one of the proposed mechanisms of action is acting as a PL pro inhibitor, same as ginger (89). Yet, in the case of turmeric (curcumin), this is not the only known antiviral mechanism; it is also known to act as an ACE II inhibitor. As previously illustrated, the virus enters the host by its S protein binding to the ACE II receptor. ACE II expression is detected in nasal epithelial cells, alveolar epithelial type II cells (AEC type II) of lungs, and the luminal surface of intestinal epithelial cells. Consequently, it stops viral entry and invasion in these cells (176–178).

#### Garlic

*Allium sativum* or garlic is one of the world’s oldest cultivated plants and has developed a well-established reputation across many cultures for embodying promising therapeutic benefits (179). More specifically, garlic is famed for its immunomodulatory role. Garlic contains a wide range of active constituents such as allicin, alliin, ajoenes, vinyldithiins, and diallyl sulfide. S-allyl-cysteine, S-ally-mercapto cysteine, and N-acetylcysteine resemble organosulfur examples, but concerning the flavonoidal constituents, quercetin is the main active constituent. The sulfur-containing phytochemicals are mainly responsible for its immunomodulatory, anti-inflammatory, anticancer, antitumor, antidiabetic, anti-atherosclerotic, and cardioprotective features (180, 181). Similar to other herbal drugs mentioned above, some mild side effects could accompany the usage of garlic such as unpleasant mouth or body odor, nausea, vomiting, and diarrhea.

##### Anticancer Activity of Garlic

The anticancer properties of garlic have been well-documented in several types of neoplastic conditions such as breast, nasopharyngeal, oral, esophageal, and gastric carcinomas, which were previously reviewed in (179). Digging deeper to understand the molecular mechanism of garlic as an anticancer agent, this could be directly related to the sulfur-containing active constituents as they provide a source of hydrogen sulfide (182). In particular, our research group has recently shown the vital role of hydrogen sulfide in cancer progression in different contexts (183, 184).

##### Immunomodulatory Role of Garlic

As previously mentioned, the immunomodulatory role of garlic extracts has a well-documented property that is mainly related to the sulfur-containing active constituents as well (180, 181). There are extensive mechanisms for the immunomodulatory and anti-inflammatory actions of garlic, specifically alliin, which effectively suppresses the expression of several proinflammatory cytokines such as interleukin 6 (IL-6) and mature plasma cell 1 (MCP-1) (110). In the case of asthmatic patients, garlic has also a proven anti-asthmatic property through repressing IL-4, IL-5, and IL-13 secretion (185). Moreover, the S-allyl cysteine constituent of garlic has also proven to ameliorate MS-related pathology and relieve the associated symptoms through altering tumor necrosis-α level in the MS-mouse model (186). However, concerning SLE patients, no information was reported concerning the impact of garlic on the pathogenesis of the disease or its associated symptoms. Collectively, garlic has been proven to have several features that could provide a protective shield for high-risk autoimmune patients in the current pandemic.

##### Anti-SARS-CoV-2 Activity of Garlic

Garlic has shown potential antiviral activity against a myriad array of viruses. Its antiviral activity against SARS-CoV-2 has been estimated. It was reported to act as a chymotrypsin-like protease (3CL^pro^) inhibitor, resulting in hindering viral attachment to host cells. Such antiviral activity has been acknowledged to the alliin and quercetin constituents in the garlic (187). In the shadow of SARS-CoV-2-associated high risk of blood clots and increase in D-dimer levels that are directly proportional to mortality rate, it is important to decrease other risk factors for blood clots such as lipids, triglycerides, and cholesterol levels in high-risk patients in particular. In such context, black garlic extracts were proven to have an anti-atherosclerotic action, meaning to decrease the blood levels of total lipids, triglycerides, and cholesterol as they lower sterol regulatory element-binding protein 1 (SREBP-1C) mRNA expression causing downregulation of lipid and cholesterol metabolism (188).

#### Flaxseed

*Linum usitatissimum* or flaxseed has been known for its potential anticancer and anti-angiogenic properties against several solid and non-solid malignancies. Nonetheless, it has also been known for its promising immunomodulatory role and recent anti-SARS-CoV-2 activity. This has been associated with its high abundance of lignans and Omega 3. The most common lignin is secoisolariciresinol diglucoside (SDG).

##### Anticancer Activity of Flaxseed

Literature supports the anticancer activity of flaxseed oil and other isolated compounds from flaxseed both *in vitro* and *in vivo* (189–191). Ezzat et al. have recently validated the anticancer activity of lignin-rich fraction from flaxseed against breast cancer cell lines and mice bearing tumors as well. It was reported that lignin-rich flaxseed fractions markedly repressed vascular endothelial growth factor (VEGF) and 1-α, metalloproteinases harnessing breast cancer metastasis *in vitro* and *in vivo* (191). Moreover, it was reported to activate the caspase-3-dependent apoptosis as a mechanism of its antiproliferative activity (190, 191).

##### Immunomodulatory Role of Flaxseed

For a very long time, PUFA has been known to treat metabolic, cardiac, inflammatory, and autoimmune diseases and reduce the risk of cancers (192). Generally, omega 3 PUFA has a great immunomodulatory effect in cases of acute pneumonia and acute respiratory distress syndrome (ARDS) by reducing reactive oxygen species and pro-inflammatory cytokines, such as TNF-α, IL-1β, IL-6, and IL-8 (193, 194).

##### Anti-SARS-CoV-2 Activity of Flaxseed

Since flaxseed’s immunomodulatory role and its inhibitory impact on several cytokines that are reported to be dominant players in the cytokine storm manifested by SARS-CoV-2 patients were validated, the effect of flaxseed on SARS-CoV-2 patients was evaluated. It was found that omega 3 reduces lung inflammation caused by the SARS-CoV-2 infection by decreasing IL-6 production, extracellular signal-regulated kinases 1 and 2, COX-2 activation, and the nuclear translocation of NF-κB (144).

#### Citrus Fruits

Citrus fruits such as *Citrus sinensis* (sweet orange) are the most widely used functional food during the pandemic. This was definitely because of its highly relevant active constituents in combating SARS-CoV-2. Citrus fruits are rich in vitamin C, carotenoids, and flavanones (195). Nonetheless, even the hesperidin flavone is found in the peel and the white part (albedo) of citrus fruits (196). Hesperidin has manifold properties such as antiviral, antimicrobial, antioxidant, antitumor, antihypertensive, and immunostimulant activities (197).

##### Anticancer Activity of Citrus Fruits (Hesperidin)

Our research group has recently focused on the anticancer activity of hesperidin and its glycoside hesperetin, where we and others showed that hesperidin has potent anticancer properties against several hallmarks of breast cancer such as cellular viability, proliferation, colony-forming ability, migration, and invasion *in vitro* (198–200). Moreover, it was found to have a direct impact on the tumor microenvironment at the tumor-immune synapse through altering ICAM-1 and ULBP2 in MDA-MB-231 breast cancer cell lines.

##### Immunomodulatory Role of Citrus Fruits (Hesperidin)

Hesperidin has been recently reported to have a direct post immunomodulatory role on autoimmune patients. It was reported that hesperidin can reduce the neuroinflammation episodes experienced by MS victims as well as ameliorates the immunological outcome in an MS-mouse model (118). In a more comprehensive study, it was reported that hesperidin alleviates several neurological disorders including MS through its anti-inflammatory and potent antioxidant activities (201). It is also important to note that hesperidin was also found to have anti-arthritogenic effects in an experimental model of RA (202).

##### Anti-SARS-CoV-2 Activity of Citrus Fruits (Hesperidin)

More than one mechanism was proposed for the anti-SARS-CoV-2 activity of hesperidin; as explained before, SARS-CoV-2 is internalized by binding of the spike glycoprotein of the virus with ACE2 receptors. Hesperidin superimposes the ACE2-receptor-bidomain (RBD) complex, so it binds to the virus spike protein (197). Also, it was suggested that it binds “3Clpro” or “Mpro”, preventing the processing of viral proteins pp1a and pp1ab into functional proteins in the host cells (145). Furthermore, it is considered a powerful antioxidant as it is powerful against superoxide and hydroxyl radicals that cause oxidative stress, and it can help control specific phases of the life cycle of SARS-CoV-2 and finally prevent cell death (197, 203–205). However, the main antioxidant effect of orange peel goes back to vitamin C content. It was suggested that increasing vitamin C daily intake during the COVID-19 pandemic is a useful protective measure as it stimulates antiviral immune responses and reduces the lungs’ inflammatory status (144, 206, 207).

#### Echinacea purpurea

*Echinacea purpurea* or the purple coneflower is a well-known herb highly recommended for respiratory infectious diseases in Europe as it is already present in different forms such as extracts, tinctures, teas, and sprays, and at different dosages as well (208). The purple coneflower contains many bioactive compounds such as chicoric acid and caffeic acids, alkylamides, and polysaccharides (209). These active constituents were proven to have antiviral effects against enveloped viruses such as human coronavirus (209). Its supplements are widely recommended by naturopathic doctors for their immune support function (210). Moreover, it is well known for its various immunomodulatory, antioxidant, anti-inflammatory, and antibacterial properties (211, 212).

##### Anticancer Activity of Echinacea purpurea

The anticancer mechanism is still not clear, but a study showed that chicoric acid has the ability to induce apoptosis in colon cancer cells and to decrease the telomerase activity in HCT-116 cells (213). Another study done on human pancreatic cancer cells and colon cancer cells indicated the ability of the root extract to induce DNA fragmentation and increase the activity of caspase 3/7 in a dose- and time-dependent manner, thus inducing apoptosis (214).

##### Immunomodulatory Role of Echinacea purpurea

Extracts of *E. purpurea*, both aqueous and alcoholic, regulate the immune cells in both adaptive and innate systems (215–217). It works by improving CD4^+^ and CD8^+^ T lymphocyte and cytokine levels in blood. These cytokines include the three interleukins IL-6, IL-10, and IL-17 (218). To inhibit inflammation, it suppresses interleukins IL-2, IL-6, and tumor necrosis factor (TNF-α) (219).

##### Anti-SARS-CoV-2 Activity of Echinacea purpurea

*E. purpurea* seems to augment its antiviral response by influencing PRRs on the innate immune cells, pathogen-associated molecular pattern PAMPs on the virus (220). Such interaction triggers phagocytosis and initiation of other antiviral responses by the immune system (221). It was also noted that *E. purpurea* has an effective antiviral role against rhinoviruses (222), influenza virus (223), RSV (208), herpes virus (208), adenoviruses (208), and coronaviruses (224, 225).

#### Java Turmeric

Another potential candidate is Java turmeric, also known as *Curcuma zanthorrhiza*. It is a highly promising candidate as its major active constituent is xanthorrhizol (accounts for 44.5%) (226). Java turmeric is widely used in Southeast Asian countries and belongs to Zingiberaceae and *Curcuma* genus (90). This plant is usually used as an important food additive to enhance flavors (227), but as a treatment component, it is well approved for some diseases and can be used as supplements (226–229). This plant has a myriad of functions, namely, it has antimicrobial, antioxidant, antihyperglycemic, antihypertensive, antiplatelet, anticancer, and nephroprotective effects, and it can be used as a supplement in SLE (230–233). These characteristics make it a potential adjuvant therapy for COVID-19 patients and a preventive measure for high-risk patients, especially SLE patients.

##### Anticancer Activity of Java Turmeric (Xanthorrhizol)

The anticancer activity of Java turmeric can be due to the induction of the TP53-dependent mitochondrial pathway and thus induction of apoptosis (234–236). It can also induce caspase activation, which will lead to enzymatic proteolysis of DNA and cytoplasmic proteins leading to cell death (227). A study done on HCT166 colon cancer showed that xanthorrhizol leads to higher expression of NAG-1 and increases the activity of its promoter (237). NAG-1 (non-steroidal anti-inflammatory drug-activated gene 1) is a pro-apoptotic and is a member of (TGF-β) (237). Regulation of the MAPK pathway is another function for xanthorrhizol; it increases ROS levels intracellularly and enhances phosphorylation of p38 and JNK in SCC-15 oral squamous cell carcinoma (238).

##### Immunomodulatory Role of Java Turmeric (Xanthorrhizol)

It was proven that it inhibits the production of inflammatory cytokines from adipose tissue by downregulation of inflammatory cytokine genes and inhibits the expression of TNF-α as well (90). For SLE patients with hypovitamin D levels, a study showed that xanthorrhizol can lower the serum level of IL-6 and increase the serum level of TGF-β (229). Another study released the same results when done on hippocampal neurons and primary culture microglia, and this inhibition of inflammation could be due to inhibition of nitric oxide synthase (iNOS), and consequently, lower levels of nitric oxide (NO) are produced (235, 239, 240). Collectively, it may play an immunosuppressant role (90).

##### Anti-SARS-CoV-2 Activity of Java Turmeric (Xanthorrhizol)

Xanthorrhizol was shown to have a potent antiviral activity against SARS-CoV-2 variants such as GH clade strain and delta strain, so it can be a promising antiviral plant against COVID-19 (241).

#### Ashwagandha

*Withania somnifera* is widely known for its antiviral, immunomodulatory, anti-inflammatory, anti-stress, antihypertensive, and antidiabetic effects, and many clinical trials were made to study its safety profile in humans (which eventually confirmed its safe use in humans) (242, 243). Moreover, there are scientific proofs for the ability of *W. somnifera* to maintain immune homeostasis in states of infection and inflammation (244, 245). The main active constituent in this plant is called Withanolides, which is a group of C28 steroidal lactone triterpinoids, including withaferin A; withanolide A, B, and D; withanoside IV and V; withasomniferin A; withanone; sitoondoside IX and X; and 12-deoxywithastramonolide. Furthermore, there are other active constituents such as catechin, naringenin, and syringic acid p-coumarin. This combination of such significant components endows *W. somnifera* superior protective capability (245, 246).

##### Anticancer Activity of Ashwagandha

Withaferin A is considered to be the principal component in *W. somnifera*; it works by inhibition of β-tubulin and consequently stops the proliferation of cells (247); it also inhibits tumor proteasomal chymotrypsin (248). It was also proven that withaferin A inhibits the cancer chaperon Hsp90 as it stabilizes the signaling proteins (249). Moreover, Notch1, which mediates the survival of colon cancer cells, is inhibited as well by withaferin (250).

Collectively, withaferin A and withanone promote ROS signaling, so they induce cancer killing by oxidative stress along with other pathways (251, 252). Finally, a study was carried out in a mouse model that concluded that *W. somnifera* alcoholic extracts inhibit tumor proliferation and growth and increase life span (253).

##### Immunomodulatory Role of Ashwagandha

Withanolide A encourages B- and T-cell proliferation with improvements in TH1 response as well (254–258). In mice, *W. somnifera* extracts led to higher counts of leukocytes and platelets (259, 260), and in chicks, the count of CD4^+^ and CD8^+^ also increased when compared to normal levels (261, 262). *W. somnifera* extracts were found by a study to be an immunostimulant when administered with anupana as vehicle, and the results revealed the activation of T cells and NK cells after 4 days only with BID consumption (263).

##### Anti-SARS-CoV-2 Activity of Ashwagandha

*W. somnifera* can impede the viral replication cycle. Withanone destabilizes the complex between the ACE2 receptor (host) and spike protein (virus) (264), and in addition, withaferin A and withanone are responsible for blocking Mpro and TMPRSS2 enzymes, which could interfere with the entry of the virus (142, 264, 265). Withacoagin and withanolide B have the ability to block the spike protein and also the RdRp with a high affinity (266). It was reported that they prevent virus entry to the host through inhibition of the trans-membrane protease serine 2 (TMPRSS2)/ACE II complex, thus hindering SARS-CoV-2 entrance to host cells (142).

#### Green and Black Tea

Tea is from the plant *Camellia sinensis*, which is a highly consumed beverage worldwide, with approximately 2.5 million tons produced each year. The difference between green and black tea is in the manufacturing process as green tea, once harvested, is steamed to prevent fermentation, while black tea is left as it is, causing the dimerization of catechins to theaflavins (267, 268). Although the composition of tea can change according to the climate, leaves, season, etc., the main constituent in it is considered to be polyphenols (269). Tea is not just a normal beverage, as research has turned a spotlight on it to study its various effects whether *in vivo* or *in vitro* (269). The studies revealed that polyphenols present in the tea can have a role in several diseases including cancer, diabetes, and cardiovascular diseases (269).

##### Black Tea

The polyphenols present in black tea are mainly theaflavins and thearubigins (269). Derivatives of theaflavins are theaflavin (TF1), theaflavin-3-gallate (TF2A), theaflavin-3’-gallate (TF2B), and theaflavin-3,3’-digallate (TF3) (270). Investigations of their biological properties found a myriad of benefits including antiviral, anti-inflammatory, antioxidant, antitumor, and antibacterial activities (271–273).

###### Anticancer Activity of Black Tea

Black tea shows a potential in the treatment of many types of cancer such as breast, prostate, lung, ovarian, cervical, and liver (274). *In vitro* studies for breast cancer showed 40% smaller tumor size for the intervention group when compared to controls (275). For prostatic cancer, significant inhibition for the androgen receptor promoter region along with inhibition of androgen receptor expression was noticed (276). Moreover, prostatic adenocarcinoma cell viability is inhibited in a dose-dependent fashion with TF1, TF2a, TF2b, and TF3 (277). A myriad of studies have proven the antiproliferative activity of theaflavins and the inhibition of survival and migration ability of cancer cells (274). There are studies that reveal the proapoptotic potential of theaflavins by observing higher levels of Bax (apoptotic protein) and lower levels of Bcl-2 (antiapoptotic protein) (274). Furthermore, P53 levels are increased by theaflavins and reduction in the levels of phosphorylated Akt, phosphorylated mTOR, and c-Myc occurs (274). Generally, theaflavins show a potential for cancer treatment and prevention (274).

###### Immunomodulatory Role of Black Tea

Theaflavins were proven to have the potential for inhibition of not only lipopolysaccharide (LPS)-induced intracellular adhesion molecule (ICAM)-1 but also the expression of the vascular cell adhesion molecule (VCAM)-1 by blocking pathways of NF-kB and c-Jun N-terminal kinase (JNK); this in turn will shut down the neutrophils since ICAM-1 and VCAM-1 are expressed on the endothelial cell surfaces (278–280). Theaflavins also have the capability to inhibit ROS and neutrophil elastase enzyme (the one that increases the permeability of alveolar epithelium) in a promising way (280–282).

###### Anti-SARS-CoV-2 Activity of Black Tea

The antiviral activity of black tea comes from TF1, TF2a, TF2b, and TF3, which were proven to have high affinity for 3CLpro and inhibit it (270). Theaflavins also showed a potential for inhibiting RNA-dependent RNA polymerase RdRp (283) and RBD in the spike at locations near the contact between ACE2 and spike protein (270). The roles of theaflavins go beyond treatment since TF3 was found to be able to bind to the ACE2 receptor, thus preventing spike RBD from attaching (284), leading to prophylaxis effects (285).

##### Green Tea

**Figure 3:**
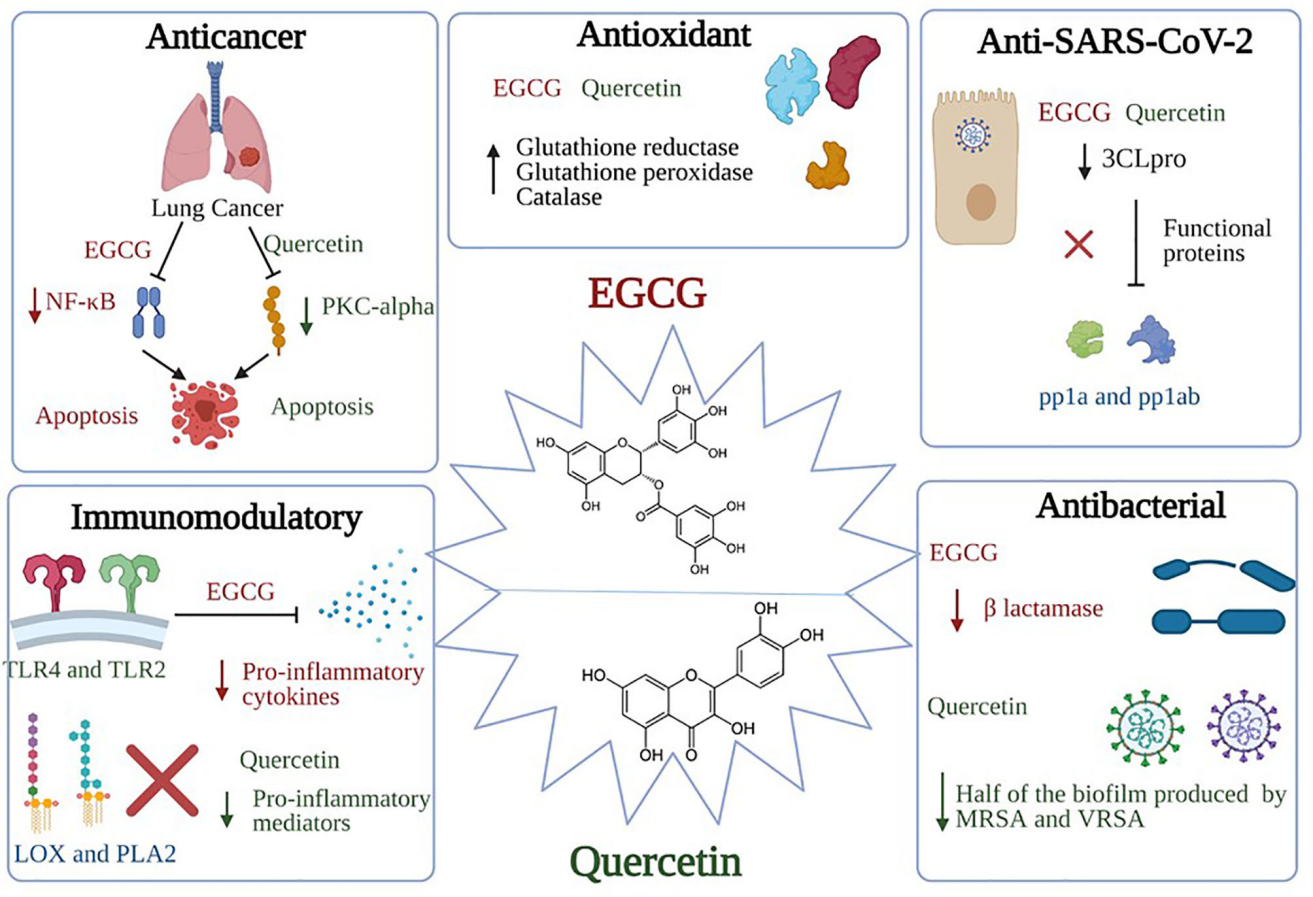
Significant pharmacological activities of EGCG and quercetin. Green tea, with its main two active constituents EGCG and quercetin, contributes to a wide range of medicinal activities such as antioxidant, immunomodulatory, anticancer, antiviral, and antibacterial actions. EGCG anticancer activity is produced by suppressing the NF-κB signaling in A549 and H1299 cells; activation of apoptotic cascades are also initiated, resulting in a marked hindering of cellular proliferation. On the other hand, quercetin inhibits the protein kinase C (PKC-α), a survival signaling protein, repressing several cancer hallmarks. Concerning the immunomodulatory actions, EGCG induces TLR4 and TLR2 expression levels, thus depleting the mitogen-activated protein kinase (MAPK) pathway and repression of pro-inflammatory cytokines release. As for the quercetin, it inhibits pro-inflammatory mediators such as Lipoxygenase and Phospholipase A2. Besides, both constituents share the same antiviral mechanism against SARS-CoV-2, which is binding to “3Cl^pro^” or “M^pro^”, preventing the processing of viral proteins pp1a and pp1ab into functional proteins in the host cells. Bona fida, the antioxidant effects of both EGCG and quercetin are mediated through scavenging and neutralizing the free radicals and boosting the enzymes that are responsible for detoxification such as glutathione reductase, glutathione peroxidase, and catalase. Finally, quercetin showed potential antibacterial activity through inhibiting half of the biofilm production by methicillin-resistant *S. aureus* (MRSA) and vancomycin-resistant *S. aureus* (VRSA). Similarly, EGCG showed antibacterial activities through inhibiting the B lactamase production and neutralizing the released toxins.

The main polyphenolics in green tea are quercetin and catechins, which include epigallocatechin-3-gallate (EGCG) (the most predominant one), epigallocatechin, epicatechin-3-gallate, epicatechin, gallocatechins, and gallocatechin gallate (269, 286). The main investigated biological effects were anti-inflammatory, antibacterial, antioxidant, antiproliferative, and antitumor (267), as shown in **Figure 3** and briefly described below. The main prominent effect for EGCG is being a potent antiviral more than the chemically synthesized drugs (270).

Regarding the scope of the review, their antiviral activity and their potential as anti-SARS-CoV-2 nutraceuticals will be the main focus especially since the anticancer activity and immunomodulatory role of EGCG and quercetin have been validated and previously reviewed (287, 288). This part will discuss some of the proposed mechanisms of EGCG (the main catechin) as an anticancer and immunomodulatory constituent of green tea as an introduction for its significance, then the spotlight on it, as well as on quercetin, will be turned on again in the matcha part.

###### Anticancer Activity of Green Tea

Apoptosis is the needed end result in the treatment of any cancer, so highlight was thrown on EGCG’s ability to induce apoptosis. Studies showed its ability to induce apoptosis by generating ROS and activating caspase-3 and caspase-9. Consequently, this leads to cycle arrest at the G1 phase (289, 290). NF-kB, which has a major role in apoptosis inhibition in cancer (291), was inhibited by EGCG in breast cancer, lung cancer, and human non-squamous cell carcinoma (292, 293). Activator protein-1 (AP-1), which induces proliferation, is also downregulated by EGCG (294). Actually, EGCG was proven by several studies to inhibit VEGF production through inhibition of STAT-3 and NF-kB in breast and human non-squamous cell carcinoma (292). Another study indicated the efficacy of EGCG on the inhibition of the IGF/IGF-1R axis (295, 296). Even in epigenetics, EGCG inhibits the activation of DNA methyltransferase, leading to restoration of silenced tumor suppressor genes as an end result; these genes include retinoic acid receptor- β (RARβ), p16INK4a, and O^6^-methylguanine-DNA methyltransferase (297).

###### Immunomodulatory Role of Green Tea

In an aim to investigate the effects of EGCG on cytokine level modulation, a study was done on activated human primary T cells to see the effect on atherogenesis (298). This study found that EGCG has successfully decreased the level of interleukins IL-2 and IL-4, INF-γ, and TNF-α. EGCG also decreased the level of phosphorylated c-Jun N-terminal (p-JNK) and extracellular signal-regulated kinase (p-ERK), and this could explain the mechanism used by EGCG to exert its anti-inflammatory effects (298). In addition, EGCG seems to have a role in symptoms reduction and pathology improvement in autoimmune diseases (299). Inhibition effects of EGCG on CD4^+^ T-cell expansion in response to stimulation was observed (299). The differentiation of naïve CD4^+^ T cells and that of Th1 and Th17 was also affected (299). This obstructed differentiation of Th1 and Th17 can be due to downregulation of transcription factors by EGCG, for instance, STAT1 and T-bet for Th1, while STAT3 and RORγt for Th17 (299). A study on multiple sclerosis in an animal model showed that EGCG weakened the disease severity in a dose-dependent manner and suppressed the proliferation of T cells along with reducing pro-inflammatory cytokine production (299). Besides, EGCG anti-inflammatory effects were proven as well in inflammatory arthritis disease (299).

EGCG has the ability to downregulate MAPK and NF-kb signaling pathways leading to the inhibition of pro-inflammatory cytokines as a result (300, 301). EGCG can weaken the transmigration of neutrophils through vascular endothelial cells (281) and decrease the neutrophil elastase enzyme, which increases the permeability of alveolar epithelium (302). As mentioned in black tea, EGCG in green tea can also inhibit LPS-induced ICAM-1 as well as the expression of the VCAM-1 by blocking pathways of NF-kB and c-Jun N-terminal kinase (JNK); this in turn will shut down the neutrophils since ICAM-1 and VCAM-1 are expressed on the endothelial cell surfaces (278–280). Last but not least, EGCG can scavenge for ROS and neutrophil elastase enzyme (the one that increases the permeability of alveolar epithelium) in a promising way (280–282), making it a strong immunomodulatory agent that can help fight infections that consequently will have an impact on controlling COVID-19.

###### Anti-SARS-CoV-2 Activity of Green Tea

**Figure 4:**
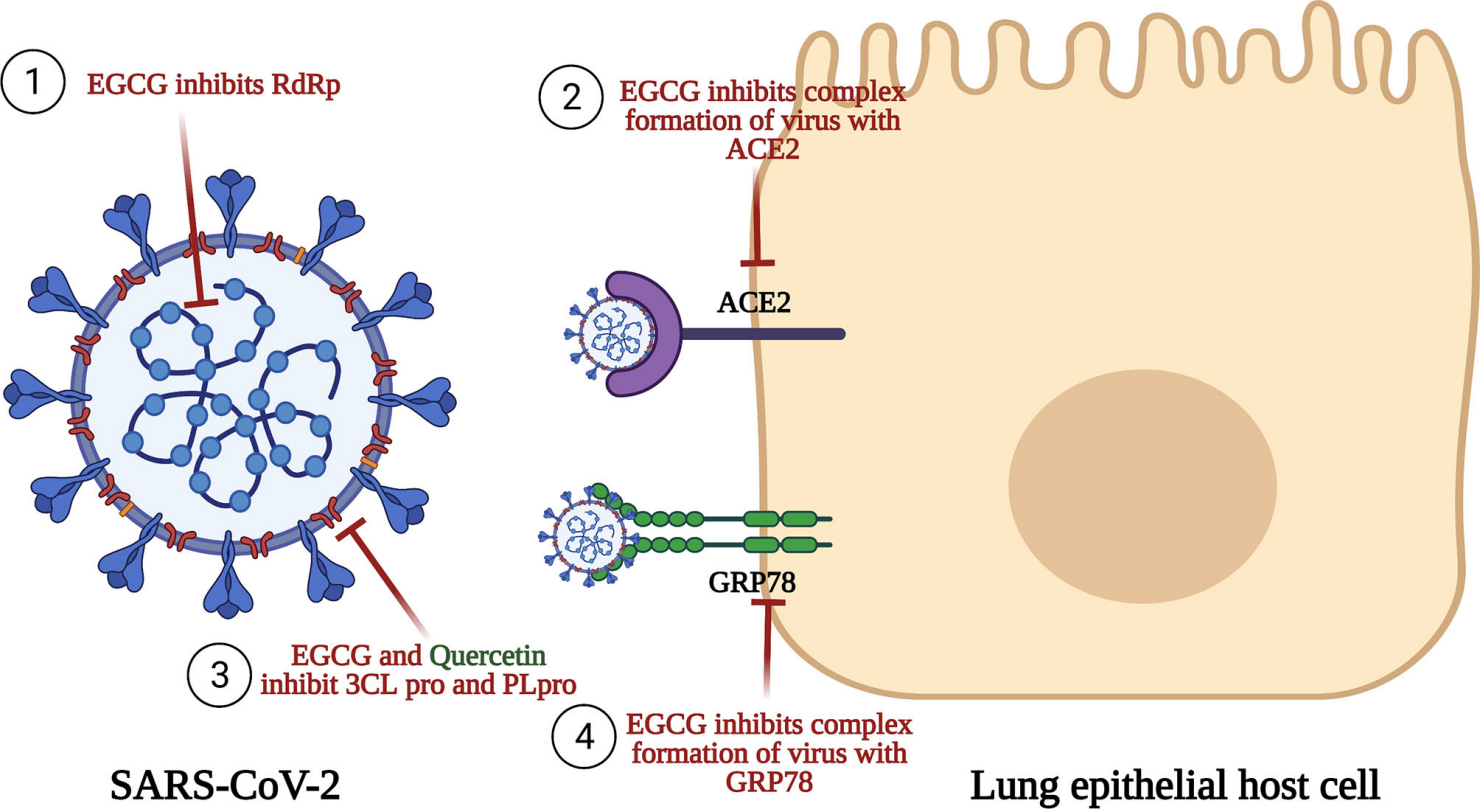
Anti-SARS-CoV-2 activities of EGCG and quercetin. This figure illustrates the anti-SARS-CoV-2 for both EGCG and quercetin. EGCG inhibits RNA-dependent RNA polymerase (RdRp), an enzyme having an important role n replication and transcription of the virus. EGCG also inhibits the binding of the S1 region of the viral spike protein to the ACE2 receptor on the lung host cells. EGCG and quercetin prevent the processing of polyproteins pp1a and pp1ab by Papain-like protease (PL^pro^) and Chemotrypsin-like protease (3C like protease or 3CL^pro^ or M^pro^). Finally, EGCG inhibits complex formation of the virus with GRP78 receptor and thus inhibits viral entry.

EGCG was shown to possess antiviral activity against many viruses such as porcine reproductive and respiratory syndrome virus (PRRSV), hepatitis C virus (HCV), ZIKA virus, chikungunya virus, influenza virus, and HIV-1 (303–307). Consequently, this inspired researchers to evaluate its antiviral potential against SARS-CoV-2. Initially, EGCG and quercetin were reported to be among the most effective inhibitors for 3CLpro as presented in **Figure 4** (148). EGCG was proven by molecular docking studies to be the most potent inhibitor for 3CLpro among all the nature-based phytochemicals (308). Then, it was reported that EGCG inhibits many structural proteins such as the HR2 domain, the post-fusion core of the S2 subunit, S protein, the RBD-ACE2 complex, and NSP15 endoribonuclease as shown in **Figure 4** (270). Also, another mechanism of action mediated by EGCG was the inhibition of the complex formation between glucose-regulated protein-78 (GRP-78) and the virus (309), as shown in **Figure 4**. GRP78 is a chaperone protein that is normally expressed in the lumen of the endoplasmic reticulum. Under cell stress conditions, overexpression of this protein occurs and is then translocated to the plasma membrane where SARS-CoV-2 interacts with it by the S protein, and subsequently, virus entry happens (310). Another molecular docking study was made on the binding affinity to the viral structural protein finding that EGCG has the highest affinity among the other substances that are included in the study. This study underlined a very important discovery: the affinity of EGCG to inhibition was higher than that of the well-known drugs used during the pandemic, remdesivir and chloroquine, suggesting a better antiviral activity for EGCG (270, 311).

Collectively, among all the natural active constituents isolated from phytochemical plants, EGCG and quercetin showed an exceptionally potent antiviral activity harnessing the SARS-CoV-2 life cycle through a myriad of mechanisms as summarized in **Figure 4**. Accordingly, our next step was to screen for herbal drugs that were reported to contain the highest phenolic contents of EGCG and quercetin. Undoubtedly, the choice was matcha, especially since it has been recently reported that several types of green tea could effectively block infection due to SARS-CoV-2 and its new variants by mainly abrogating the spike binding to the ACE2 receptor (29, 30).

#### Matcha: Filling the Gap During the Pandemic

Matcha powder is a herbal drug that was reported to contain at least three times higher EGCG content than green tea, providing an economic and beneficial beverage for SARS-CoV-2-infected patients and a preventive measure for high-risk patients such as cancer and autoimmune patients (312). Nowadays, matcha tea powder is widely known and used for its abundant health benefits and its exceptional quality. Matcha is the powdered form of green tea that originated in Japan (313). The high nutritional benefits of matcha come from the presence of many powerful active constituents as listed in **Table 4** below. The main forms of catechins and the most active ones found in higher amounts are (-) epigallocatechin 3-gallate (EGCG), caffeine, quercetin, phenolic acids, rutin, vitamin C, chlorophyll, and theanine (313). Catechins are present in four types: (-) epicatechin (EC), (-) epicatechin 3-gallate, (-) epigallocatechin (EGC), and (-) epigallocatechin 3-gallate (EGCG) (344, 345).

**Table 4:** Biological activities of matcha active constituents.

Active constituent	Biological activity	References
Chlorophyll	Anti-inflammatory Antioxidant	(314, 315)
Epigallocatechin 3-gallate (EGCG)	Decreases the ROS Increases enzymes for detoxification Anti-carcinogenic Anti-bacterial Antiviral Immunomodulatory	(300, 316–319)
Quercetin	Antioxidant Neuroprotective Decreases glucose absorption Increases insulin secretion and sensitivity Antiviral Immunomodulatory Anti-bacterial Anti-carcinogenic	(320–331)
Vitamin C	Strong exogenous antioxidant Enforces the immune system	(332)
Caffeine	Decreases the ROS Increases the antioxidant enzymes activity Increases glutathione levels Decreases pro-inflammatory cytokines	(333, 334)
Theanine	Gives distinctive and non-bitter taste	(335–337)
Phenolic Acids	Antioxidant Anti-inflammatory Hypoglycemic Neuroprotective Regulates carbohydrates/lipid metabolism	(333, 338–341)
Rutin	Antioxidant Anti-diabetic Anti-inflammatory	(332, 342, 343)

#### Matcha Main Constituents: EGCG

Catechins have an indisputable role as antioxidants by scavenging and neutralizing the free radicals and boosting the enzymes that are responsible for detoxification such as glutathione reductase, glutathione peroxidase, and catalase (346). It is also worth mentioning that the cellular redox homeostasis can be well maintained by the intake of catechins more often in the human diet (316). As previously mentioned, EGCG has a powerful antiviral effect. Compared to vitamin C, flavonoids, and glutathione, it has been proved that catechins have a higher antioxidant potential (316).

##### The Anti-Carcinogenic Activity of EGCG

**Figure 5:**
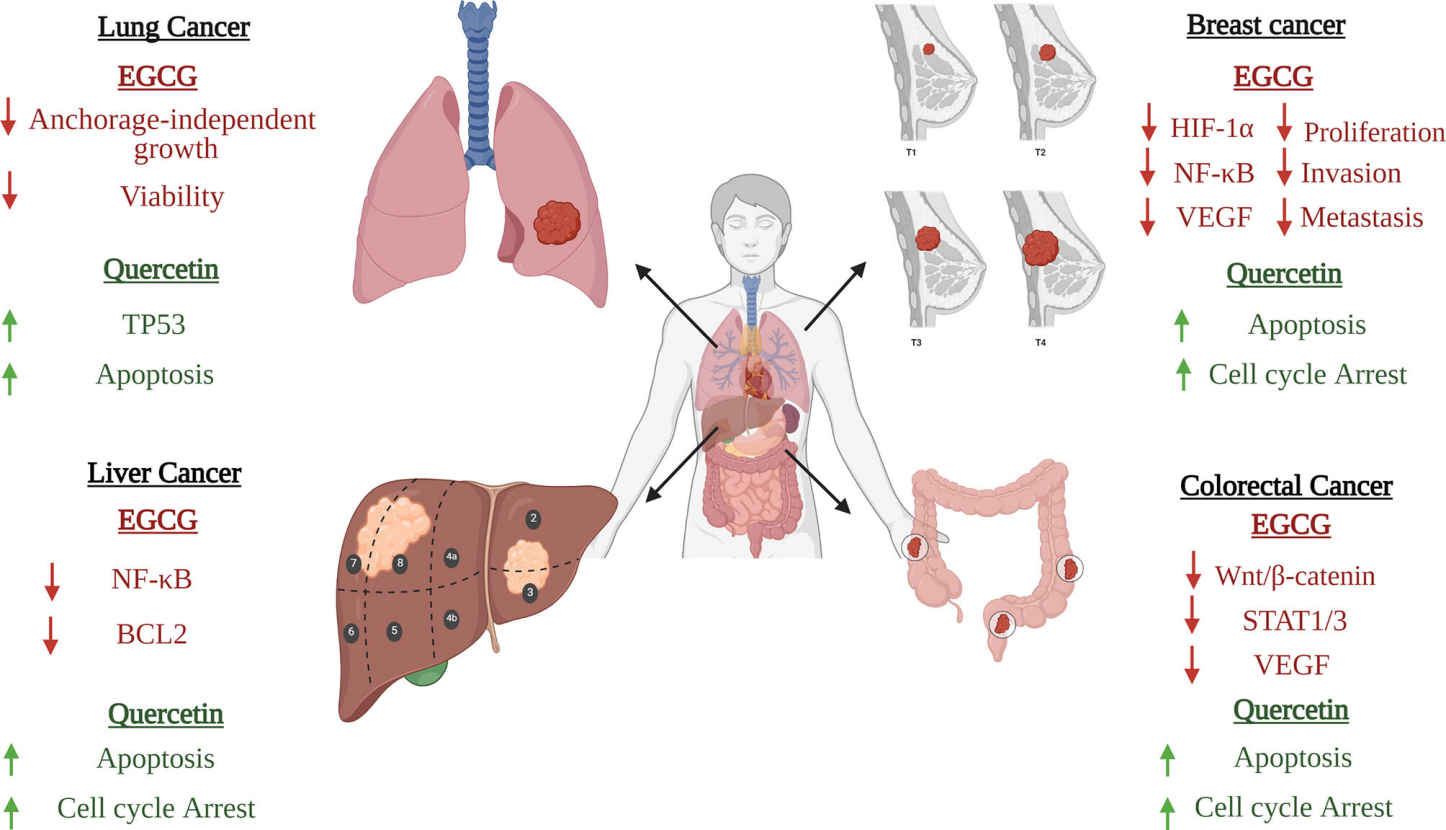
Anticancer activities of EGCG and quercetin. Epigallocatechin-3-gallate (EGCG) and quercetin modulate several canonical oncological pathways such as Wnt/β-catenin and JAK/STAT pathways. Also, they modulate the expression of several anti-apoptotic proteins such as BCL2, tumor suppressor proteins such as TP53, and oncogenic drivers such as VEGF. Both active ingredients have proven to be effective in halting the malignant transformation process in several types of cancers such as breast, lung, liver, and colorectal cancers.

This antioxidant effect contributes to one of the anti-carcinogenic mechanisms of EGCG, as it was previously mentioned that the catechins can quench the reactive oxygen species at any stage and consequently halt the malignant transformation process (347). Other studies have shown that the EGCG has several anticancer activities as shown in **Figure 5**. EGCG exhibits antitumorigenic properties in lung cancer. It suppresses the NF-κB signaling in A549 and H1299 cells; this leads to the inhibition of cell proliferation and induces apoptosis as shown in **Figure 5** (348). EGCG suppresses breast cancer progression through the tight binding of EGCG to signal transduction activator proteins of transcription 1 (STAT1) by its three hydroxyl groups of the B ring and one hydroxyl group of the D ring; this bond leads to the blockage of the phosphorylation of STAT1 by Janus Kinase 2 (JAK2) and inhibition of its carcinogenic effects since STAT1 in cancer cases can act as an oncogenic protein. It was worth noting that EGCG promotes Fas/CD95-mediated apoptosis in the neck and head squamous carcinoma by inhibiting JAK/STAT3 (317, 349).

##### Anti-Bacterial Activity of EGCG

As per the current treatment protocol for SARS-CoV-2, several antibiotics are prescribed especially in high-risk patients to avoid the complications of secondary bacterial infections, an act that would result in an antibiotic resistance catastrophe post-pandemic as described by the WHO in November 2020. As presented in **Figure 3**, EGCG has promising antibacterial activity while saving the world from the antibiotic resistance dilemma that would threaten our lives enormously. EGCG has shown bactericidal activity against staphylococci. Moreover, EGCG also shows an antibiofilm activity when co-administered with other antibiotics; it gives a powerful synergistic action. EGCG also can inhibit beta-lactamase production and neutralize the released toxins. The negatively charged property of EGCG makes it more effective against Gram-positive bacteria (318).

##### Immunomodulatory Role of EGCG

Nutrition immunity is a new concept that has been revolutionized during the pandemic (350). Massive attack on the respiratory epithelium (host cells for SARS-CoV-2) can lead to acute respiratory distress syndrome (ARDS), characterized by the uncontrolled release of pro-inflammatory cytokines leading to damaging the host cells, a vicious process termed as cytokine storm (350). It is important to note that the cytokine storm is also a common feature in the case of chemotherapy-treated cancer patients (351). Therefore, it is a crucially important measure to protect such high-risk patients during the pandemic. SARS-CoV-2-infected patients have a reduction in IFN-α and IFN-β levels, thus increasing the chance for the virus to invade and take over the immune system (319, 352). It is noteworthy that EGCG has a dominant immunomodulatory role by inducing TLR4 and TLR2 expression levels. Such induction occurs as a result of the repression of the mitogen-activated protein kinase (MAPK) and the pro-inflammatory cytokines as presented in **Figure 3** (300, 301). EGCG also modulates the immune system through inhibition of the RIG-I (acts as a RIG-I inhibitor), thus protecting the infected patient from the cytokine storm and its notorious consequences (353).

#### Matcha Main Constituent: Quercetin

As presented in **Table 4**, one of the main matcha constituents is quercetin. Quercetin possesses an array of pharmacological activities such as neuroprotection, antioxidant, and antineoplastic activities. It also has a vital role in diabetes mellitus patients, where it inhibits glucose absorption and thus it regulates carbohydrate metabolism, thus regulating the insulin secretion and sensitivity to tissues (320, 321, 354).

##### Anticancer Activity of Quercetin

Our research group has recently highlighted the potential anticancer activity of quercetin and its derivatives in liver and breast cancers (355, 356). Studies show that if quercetin was ingested on a daily basis, it was found to decrease the risk of cancer incidence (326). Quercetin was also reported to retain its antitumorigenic properties against several types of leukemias, melanoma, lung, colorectal, and ovarian cancers (325). *In vivo* studies also supported the promising anticancer properties of quercetin in several animal models (324). Molecularly, quercetin inhibits protein kinase C signaling protein, resulting in the activation of apoptotic death signals and cell cycle arrest as shown in **Figure 5** (329). It also has powerful induction effects on TP53, Fas/FADD, caspases, and suppression of vital anti-apoptotic proteins (330, 331, 357). From an immune-oncological point of view, long-term intake of quercetin was proved to improve natural killer cells’ cytotoxic activity, neutrophil chemotaxis, and lymphocyte proliferation (323, 358). In addition, quercetin induces T helper cells to produce TH1-derived interferon-gamma (IFN-γ) and downregulates TH2-derived IL-4 (327, 359). Altogether, it is quite evident that quercetin possesses potential intrinsic anticancer activity together with activating the innate and adaptive immune arms to halt oncological progression in several malignant contexts.

##### Antibacterial Activity of Quercetin

Similar to EGCG, quercetin has shown potent bactericidal properties against an array of bacteria, such as *Enterococcus faecalis* and *Listeria monocytogenes*. Both are resistant to several antibiotics and have a detrimental ability to produce biofilms on an artificial device such as stents. Quercetin was reported to effectively inhibit 95% of biofilm formation, also by stopping several glycolytic enzymes such as 2,3-bisphosphoglycerate-dependent phosphoglycerate mutase (GpmA) and ATP-dependent phosphofructokinase (PfkA) in *L. monocytogenes*. Quercetin also represses the secretion of the bacterial adhesion molecules that have a vital role in *L. monocytogenes* (foodborne illness bacteria) infection. Thus, the incorporation of quercetin as a food additive to minimize the adhesion, proliferation, and biofilm growth of the bacteria is a safe and economic idea (328). It is also important to note that quercetin was reported to inhibit half of the biofilm production by methicillin-resistant *Staphylococcus aureus* (MRSA) and vancomycin-resistant *S. aureus* (VRSA), thus shedding light on its powerful antibacterial activity (328).

##### Immunomodulatory Role of Quercetin

Quercetin possesses an immunomodulatory role through repressing platelet aggregation, lipid peroxidation, inhibition of pro-inflammatory mediators such as lipoxygenase and phospholipase A2, and the expression levels of MHG class II and co-stimulatory molecules. Digging deeper, it was found that attenuation of several canonical and non-canonical immunomodulatory pathways such as arachidonic acid metabolism, the associated leukotriene/prostaglandins, and mTOR signaling pathways are the molecular mechanisms by which quercetin possesses its immunomodulatory role in several contexts (360, 361).

#### Matcha Other Constituents: Caffeine

Caffeine is one of the constituents of matcha; it has a strong antioxidant activity where it acts by neutralizing the ROS and it induces the antioxidant enzyme activities and also increases the glutathione levels, thus reducing oxidative stress. Besides the antioxidant effect, caffeine also has an anti-inflammatory activity where it reduces the secretion of pro-inflammatory cytokines. It was found that the caffeine content in matcha is greater than that in green tea, thus making matcha tea more effective (333, 334, 339).

#### Matcha Other Constituents: Phenolic Acids

Phenolic acids are found at their maximum levels in matcha tea. They are well known to have powerful antioxidant and anti-inflammatory effects as well as hypoglycemic and neuroprotective effects. Moreover, regulation of several metabolic disorders is controlled by some of the phenolic acids by regulating carbohydrates and lipid metabolisms (333, 338–341).

#### Matcha Other Constituents: Rutin

Rutin is a polyphenolic compound. Among all the kinds of tea available in the market, matcha contains very high amounts of rutin. It has several benefits such as antioxidant, antidiabetic, and anti-inflammatory effects (332, 342, 343, 362).

#### Matcha Other Constituents: Chlorophyll, Theanine, and Vitamin C

Chlorophyll is responsible for the bright green color of matcha. It was reported that it has powerful anti-inflammatory and antioxidant effects (314, 315). Nevertheless, the amino acid theanine provides the taste of matcha, which is distinctive and non-bitter. Also, it was found that the presence of caffeine with theanine improves efficiency rather than using them separately (335–337). Last but not least, the presence of vitamin C is also important and possesses several beneficial effects since it cannot be synthesized within the human body. It is considered a strong exogenous antioxidant, and it must be supplied *via* nutritional intake as it reinforces the immune system (363).

## Discussion

COVID-19 has made the world face a war with a different meaning this time—a war in which the entire world population are warriors, whose main slogan was “Stay Safe”, a war whose weapons consist of open-ended practical trials that take place in research labs to find a solution to finally end the war. On one hand, the hospitals were at full capacity, and the demand for oxygen supplies was increasing. On the other hand, this virus impacted the entire globe with deleterious effects economically. The mess was escalating.

The trials for combating this virus were numerous, and carried out with different aims, whether for drug repurposing or trying to develop new antiviral agents. Vaccine development was also one of the main goals of researchers, yet many other researchers have shed light on the use of herbal medicine. *In silico*, *in vivo*, and *in vitro* studies were conducted all with only one aim, which is to find a solution to solve this mess. In fact, none of the studies could underestimate the significance of the others, and all can work in harmony with each other or help each other to reach the main curative goal at the end.

Herbal medicine was one of the major routes that were investigated throughout this pandemic by many researchers as plants have been proven to be a miracle drug throughout the generations for combating many diseases, which also gave many researchers hope for defeating COVID-19. Nature has never failed to protect us, that is why the first routine that was followed since the start of this pandemic is to eat fruits and vegetables because of their potential to strengthen our immune system and act as a preventive measure. For this reason, it was not surprising that many review articles, clinical trials, and molecular docking studies investigated the antiviral potential of many plants against SARS-CoV-2, and many of them showed a strong potential to improve the pandemic situation as previously reviewed in (364–366).

However, there was another dark side to the story, and this darkness relies mainly on the ones who were suffering every minute whether for fear of catching the virus because they know how weak their bodies are to defeat this enemy or for the difficulties they would face to follow their treatment plans in the hospitals or clinics during this pandemic. These sufferers are mainly the cancer patients and immune-compromised patients such as those with SLE, RA, and MS who have higher mortality rates and exacerbated conditions upon exposure to the virus (367) compared to other normal individuals. Although vaccines seemed to be a proper solution, there are still limitations that should be taken into consideration as regards the efficacy of the vaccine for this type of patients as well as the possible interactions between both the vaccine and their treatments or the disease condition (367). Moreover, these patients needed to be tracked routinely for any signs of unexpected adverse events or if they are on active cancer therapy, so the relation between the timing of the vaccine and its safety and efficacy with the treatments and immune deficiency should be evaluated (368). Because of this, it was logical to think of herbal medicines as an option for these people due to their potential to defend against a myriad of viruses and strengthen the immune system, and certain herbs could have a role in attacking cancer as well when compared to vaccines or synthetic drugs.

In this review, we focused on the significant role of herbal medicines in helping cancer and immune-compromised patients. A spotlight was thrown on many plants such as ginger, turmeric, garlic, flaxseed, citrus fruits, *Echinacea purpurea*, Java turmeric, ashwagandha, and black tea. All of the plants highlighted in this review have proven their efficacy as anticancer, immunomodulatory, and antiviral agents; many of them already show an anti-COVID-19 potential. The combination of these three actions suggests herbal medicines as a good option for these patients. Yet, it is worth mentioning that most of the herbal products’ actions mentioned in this review are dose-dependent effects. For instance, it should be noted that garlic is an anticancer agent in several oncological contexts, but it is a source of organosulfur compounds, which are hydrogen sulfide donors (182). Hydrogen sulfide is a well-known biphasic gasotransmitter molecule that, at low concentrations, plays an oncogenic role while having an anticancer activity at higher concentration (183, 184).

One of the herbal plants that were discussed in this review was green tea, and while focusing on its constituents, which were mainly EGCG and quercetin, they were found to have very potent multiple mechanisms for defending against different cancer types, acting as immunomodulatory, anti-inflammatory, and antiviral agents specifically towards COVID-19. Matcha was able to obtain these protective properties in the highest possible amount.

**Figure 6:**
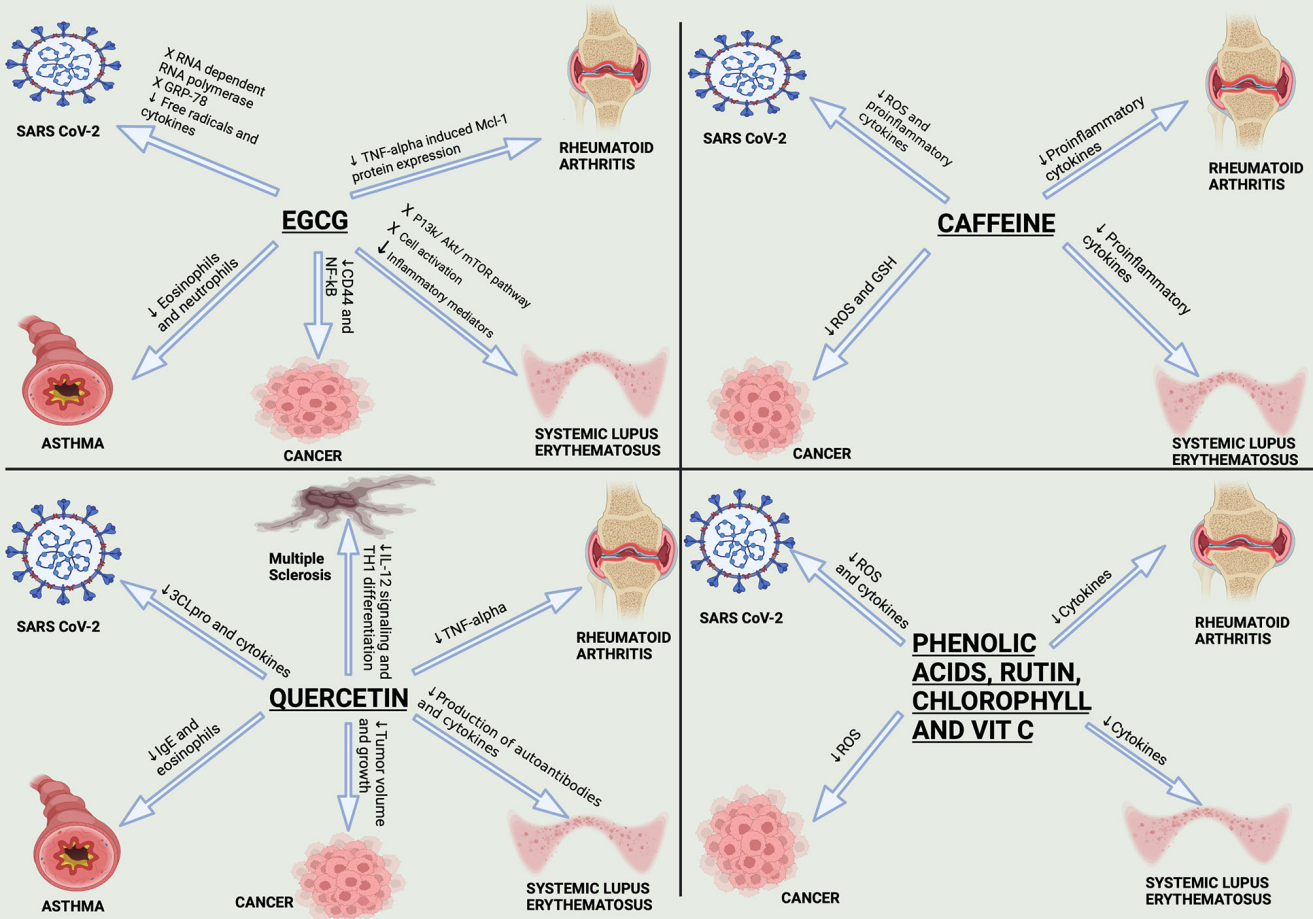
Matcha beneficial effects in protecting cancer and autoimmune patients from SARS-CoV-2 infection. This figure highlights the dynamic constituents of matcha and its beneficial effects in preventing SARS-CoV-2 infection and also ameliorating SARS-CoV-2-positive patients. A special focus on cancer and autoimmune patients is presented. Patients who have caught SARS-CoV-2 were found to have a decrease in IFNs; thus, EGCG stimulates the expression of both TLR4 and TLR2, and this helps in reducing the pro-inflammatory cytokines and the cytokine storm. Since the viral nucleic acid activates the RIG-1 that increases IFN-1, EGCG showed to act as a RIG-1 inhibitor. In addition, EGCG has an antioxidant effect by neutralizing the free radicals and boosting the detoxification enzymes. Moreover, quercetin is a potent immunomodulatory in SARS-CoV-2, as it has many functions such as antiviral activity, platelet aggregation inhibition, and inhibition of proinflammatory mediators like Lipoxygenase (LOX) and Phospholipase A2. Rutin has an antioxidant and anti-inflammatory activity; thus, it is useful in the management of COVID-19. The chlorophyll content in matcha has proven to be beneficial due to its anti-inflammatory and antioxidant effect, which assists to overcome the cytokine storm. Besides, caffeine has a potential anti-inflammatory effect by decreasing proinflammatory cytokines, and its antioxidant effect is due to reduction of ROS and increase of glutathione. In addition, Vitamin C greatly stimulates antiviral immune responses and reduces the lungs’ inflammatory state. It is essential to highlight that the intake of matcha tea not only will manage COVID-19 symptoms but also can prevent the virus itself from infecting humans.

Matcha is a Japanese green tea in which nowadays seems to be trendy in certain populations for its claimed ability to boost health and immunity, also it has been used recently in some of the cosmetic products for its ability to participate in a healthy skin conditions. Digging deeper in the Matcha constituents, we found that it contains EGCG and quercetin, the proven ones for their efficacy, in much concentrated amounts than in the normal green tea along with other constituents that were discussed as well in this review and as shown in **Figure 6**. This can explain the potential for that herbal tea in specific to be an indispensable way for cancer and immunocompromised individuals to protect themselves against COVID-19 along with alleviating their health states.

This review suggests matcha as one of the potential options that should be highlighted during this period as it offers a great amount of potential to battling the current pandemic.

## Future Recommendations

Since herbal medicines have always been able to tackle many health issues throughout the past pandemics and act as prophylaxis against a myriad of diseases, this encourages us to have a wider look in generalizing herbal medicines to the entire population. As reviewed, it has been documented that with the emergence of each new mutation, the efficacy of vaccines and drugs becomes negatively affected. Such information should encourage global health organizations to tackle this issue in a different way. More in-depth studies on herbal medicines need to be conducted using more clinical trials. *In vivo* and *in vitro* studies should be carried out for much more medicinal plants as well as *in silico* and molecular docking studies to further study and discover new effects for secondary metabolites. Such studies and clinical trials should also include the more susceptible populations such as cancer patients, immune disease patients, and children since they are not included in the current studies. Because of this, WHO will be encouraged to advise doctors about prescribing these herbal medicines along with other synthetic drugs if needed, and the media will also play a role as they will start to encourage people to use more herbal medicines and to make them aware of such medicines’ benefits. Consequently, this can lead to the discovery of more new plants and the investigation of new research areas by pharmaceutical companies in order to meet the market need. This can be a plan for any upcoming SARS-CoV-2 outbreak or for any new pandemic for either normal or ill people, and this plan will most probably succeed. One of the herbal medicines that is highlighted by this review for future use is matcha.

Such a wide range of therapeutic potentials ofwfi 2 “matcha” constituents whether as an immunomodulatory agent or as an anti-SARS-CoV-2 agent might be the reason why “matcha” would acquire a high market share in the upcoming years, especially since SARS-CoV-2 might behave like seasonal flu (after having more than eight waves to date). Nonetheless, we should not ignore the fact that more coronaviruses might appear at any time, since bats act as a reservoir or a storage tank for them. Collectively, this might highlight the potential of “matcha” to be the stone that could hit 3 birds (cancer, autoimmune disease, and SARS-CoV-2).

Although “matcha” seems to be very promising, there are a lot of challenges that may hinder its usage. For instance, “matcha” might not be accessible for many people due to its high price especially since we are focusing on cancer and autoimmune disease patients who already have very high expenses for their medications. Therefore, it is recommended to be produced in larger amounts for the sake of reducing its price, and this could be done by pharmaceutical companies. One of the major disadvantages that might discourage people to use “matcha” is its bad taste; however, this could be masked by the addition of flavors during the production phase, which again sheds light on the importance of pharmaceutical companies in “matcha” production. Therefore, this review elucidates the importance of having a cup of “matcha” to reinforce and strengthen the immune system in cancer and autoimmune disease patients who have a higher risk of catching SARS-CoV-2. Yet, this also can be generalized for everyone as it is powerful enough to prevent and protect them from catching the virus.

## Conclusion

In conclusion, this review stresses the fact that the probability of the current pandemic to continue for a long time and the probability of developing future pandemics are extremely high, especially after the emergence of several VOCs. In this review, the authors highlight the great potential held by herbal medicine especially for high-risk patients such as cancer and autoimmune patients. Also, the authors shed light onto our new norm and how herbal products are considered risk-free solutions. In this review, it was set clear that after the SARS-CoV-2 pandemic experience, it should be noted that the development of new drugs and effective vaccines will not always be the easiest option. This review presents the current herbal medicines that could be used in preventing and fighting COVID-19, which happen to have three roles: as an immunomodulatory and anticancer agent, aside from displaying anti-SARS-CoV-2 activities. A special spotlight was turned on for the Japanese green tea “matcha”. The authors elucidating the promising use of matcha as a prophylactic agent during the SARS-CoV-2 pandemic can have a significant impact on the socioeconomic and health status in general and on cancer and autoimmune patients in particular. This was mainly based on their major constituents: EGCG and quercetin and their well-reported anticancer activity, immunomodulatory effects, and their recent anti-SARS-CoV-2 activity. Yet, more detailed studies about the usage of “matcha” among cancer and autoimmune patients have to be conducted in the future.

## Author Contributions

CK, MK, and MT contributed to drafting the original draft of the manuscript and data collection (literature reviewing) and sketching the figures. The conception of the work, critical revision of the article, and data interpretation were performed by the principal investigator of the work, RY. The final version of the manuscript was approved and revised by all the authors.

## Conflict of Interest

The authors declare that the research was conducted in the absence of any commercial or financial relationships that could be construed as a potential conflict of interest.

## Publisher’s Note

All claims expressed in this article are solely those of the authors and do not necessarily represent those of their affiliated organizations, or those of the publisher, the editors and the reviewers. Any product that may be evaluated in this article, or claim that may be made by its manufacturer, is not guaranteed or endorsed by the publisher.

## Glossary

**Table:**

+gRNA	Positive single-stranded RNA
+sgRNA	Positive subgenomic RNA
2019-nCoV	2019 novel coronavirus
3C like protease	Chemotrypsin-like protease
3CLpro	Chemotrypsin-like protease
ACE II	Angiotensin-Converting Enzyme 2
Ad5	Adenovirus type 5
AKT	Protein kinase B
ALA	Alpha linolenic acid
ARDS	Acute respiratory distress syndrome
ASCO	American Society of Clinical Oncology
BAD	Bcl-2-associated agonist of cell death
Bax	Bcl-2-associated X protein
Bcl-2	B-cell lymphoma 2
Bcl-xl	B-cell lymphoma extra large
CD 44	Cluster of differentiation 44
COVID-19	Coronavirus disease of 2019
CoVs	Coronaviruses
COX-2	Cyclooxygenase 2
CYP3A4	Cytochrome P450 3A4
DADS	Diallyl disulfide
DMTs	Disease-modifying therapies
DMV	Double-membrane vesicle
dsDNA	Double-stranded DNA
E	Envelope
EC	Epicatechin
EGC	Epigallocatechin
EGCG	Epigallocatechin 3-gallate
ER	Endoplasmic reticulum
ERGIC	Endoplasmic reticulum golgi intermediate compartment
ESMO	European Association for Medical Oncology
FADD	Fas-associated protein with death domain
Fas/CD95	Cluster of differentiation 95
FDA	Food and Drug Administration
FoxP3	Forkhead box protein 3
GIT	Gastrointestinal tract
GpmA	2,3-bisphosphoglycerate-dependent phosphoglycerate mutase
-gRNA	Negative single-stranded RNA
GRP78	78-kDa glucose-regulated protein
GRP-78	Glucose-regulated protein 78
H1N1	Hemagglutinin type 1 and neuraminidase type 1
HCV	Hepatitis C virus
HE	Hemagglutinin esterase
HIV	Human immunodeficiency virus
ICAM-1	Intracellular adhesion molecule 1
ICTV	International Committee on Taxonomy of Viruses
IFN beta	Interferon beta
IFN gamma	Interferon gamma
IFN-1	Type I interferon
IgE	Immunoglobulin E
IL-10	Interleukin 10
IL-12	Interleukin 12
IL-13	Interleukin 13
IL-1α	Interleukin 1 alpha
IL-1β	Interleukin 1 beta
IL-2	Interleukin 2
IL-4	Interleukin 4
IL-5	Interleukin 5
IL-6	Interleukin 6
IL-8	Interleukin 8
iNOS	Inducible nitric oxide synthase
JAK	Janus kinase
JAK2	Janus kinase 2
LNP	Liposomal nanoparticle
LOX	Lipoxygenase
LPS	Lipopolysaccharide
M	Membrane
MAPK	Mitogen-activated protein kinase
MCP 1	Monocyte chemoattractant protein 1
MCP-1	Mature plasma cell 1
MERS-CoV	Middle East Respiratory Syndrome Coronavirus
miRNA	MicroRNA
MMP13	Matrix metallopeptidase 13
MMP9	Matrix metallopeptidase 9
Mpro	Main protease
mRNA vaccines	Messenger RNA vaccines
MRSA	Methicillin-resistant *S. aureus*
MS	Multiple sclerosis
mTOR	Mechanistic target of rapamycin
MUC 2	Mucin 2
N	Nucleocapsid
NCCN	National Comprehensive Cancer Network
NF-ĸB	Nuclear factor kappa-light-chain-enhancer of activated B cells
NIAID	National Institute of Allergy and Infectious Disease
NK cell	Natural killer cell
NO	Nitric oxide
NRVV	Non-replicating viral vector
Nsp	Nonstructural protein
Nsp1	Nonstructural protein 1
Nsp12	Nonstructural protein 12
Nsp13	Nonstructural protein 13
Nsp14	Nonstructural protein 14
NSP15	Nonstructural protein 15
Nsp2	Nonstructural protein 2
Nsp2-16	Nonstructural protein 2-16
Nsp3	Nonstructural protein 3
Nsp5-16	Nonstructural protein 5-16
Nsp7	Nonstructural protein 7
Nsp8	Nonstructural protein 8
P53	Tumor protein p53
PAF	Platelet-activating factor
PfkA	ATP-dependent phosphofructokinase
PG	Prostaglandin
PGE2	Prostaglandin E2
PH	Potential Hydrogen
PI3K	Phosphoinositide 3 kinase
PKC-alpha	Protein kinase C-alpha
PKC-delta	Protein kinase C-delta
PLpro	Papain like protease
PP	Polyprotein
PRRSV	Procaine reproductive and respiratory syndrome virus
PUFA	Polyunsaturated fatty acids
RA	Rheumatoid arthritis
RBD	Region binding domain
RdRp	RNA-dependent RNA polymerase
RIG-I	Retinoic acid-inducible gene I
RORγt	Retinoic acid-related orphan receptor γt
ROS	Reactive oxygen species
RTC	Replication/Transcription Complex
S	Spike
saRNA	Self-amplifying messenger RNA
SARS-CoV-2	Severe Acute Respiratory Syndrome-Coronavirus-2
SDG	Secoisolariciresinol diglycoside
-sgRNA	Negative subgenomic RNA
SLE	Systemic lupus erythematosus
SOCS3	Suppressor of cytokine signaling 3
SREBP-1C	Sterol regulatory element-binding protein 1
STAT	Signal Transducer and Activator of Transcription
STAT1	Signal Transducer and Activator of Transcription 1
STAT3	Signal Transducer and Activator of Transcription 3
T-bet	T-box protein expressed in T cells
TF1	Theaflavin
TF2A	Theaflavin-3-gallate
TF2B	Theaflavin-3’-gallate
TF3 or TFDG	Theaflavin-3,3’-digallate
TGF-β	Transforming growth factor beta
TH1	T helper 1 cell
TH17	T helper 17 cell
TH2	T helper 2 cell
TLR	Toll-like receptor
TLR2	Toll-like receptor 2
TLR4	Toll-like receptor 4
TMPRSS2	Transmembrane protease serine 2
TNF-α	Tumor necrosis factor alpha
ULBP2	UL16 binding protein 2
VAERD	Vaccine-associated enhanced respiratory disease
VEEV	Venezuelan equine encephalitis virus
VEGF	Vascular endothelial growth factor
VRSA	Vancomycin-resistant *S. aureus*
